# Mitigation of renal tubular injury by SIRT6 may improve individual outcomes in diabetic kidney disease-potential mechanisms involving epigenetic repression of inflammatory responses

**DOI:** 10.1016/j.jare.2025.10.008

**Published:** 2025-10-10

**Authors:** Qi Jin, Lanfang Li, Peng Qu, Fang Ma, Ping Li, Yuan Qiao, Yijia Zhang, Shuman Ran, Xinyu Li, Tongtong Liu, Liping Yang, Qian Li, Huimin Mao, Yuyang Wang, Feihong Ren, Yongli Zhan, Liang Peng

**Affiliations:** a Beijing Key Laboratory for Immune-Mediated Inflammatory Diseases, Institute of Clinical Medical Sciences, China-Japan Friendship Hospital, Beijing 100029, China; b Guang’anmen Hospital, China Academy of Chinese Medical Sciences, Beijing 100053, China; c China-Japan Friendship Hospital (Institute of Clinical Medical Sciences), Chinese Academy of Medical Sciences & Peking Union Medical College, Beijing 100730, China

**Keywords:** SIRT6, Diabetic kidney disease, Mitigation of disease progression, Targeted treatments, Epigenetic regulation, Inflammatory responses

## Abstract

**Introduction:**

Progressive tubulointerstitial injury plays a critical role in the progression of diabetic kidney disease (DKD), but the epigenetic mechanisms driving this process remain largely unclear.

**Objectives:**

This study aimed to investigate the role of the histone deacetylase SIRT6 in renal tubular epithelial cells (TECs) during DKD progression and to explore its potential as a therapeutic target.

**Methods:**

We employed digital spatial profiling (DSP) to perform spatially resolved mRNA quantification in proximal renal tubular tissue from DKD patients. Additionally, we used genetic and pharmacological approaches in DKD mouse models to assess the effects of SIRT6 deficiency or overexpression on renal injury. Mechanistic studies included RNA-sequencing (RNA-seq) and Cleavage Under Targets and Tagmentation (CUT&Tag) sequencing, which to identify SIRT6-regulated genes and epigenetic modifications.

**Results:**

Our findings revealed a significant reduction of SIRT6 in TECs from DKD patients, with its expression inversely correlating with disease severity. TEC-specific SIRT6 deficiency worsened renal injury and proteinuria in DKD mice, whereas SIRT6 overexpression or pharmacological activation provided renoprotection. Mechanistically, SIRT6 directly repressed *Nlrp3* transcription by deacetylating histone 3 lysine 9 (H3K9), thereby inhibiting NLRP3 inflammasome activation and subsequent TEC injury.

**Conclusion:**

These findings highlight SIRT6 as a protective epigenetic factor in DKD and suggest its potential utility for disease stratification, early therapeutic intervention, and precision medicine.

## Highlights

• Spatially resolved transcriptomics reveals SIRT6 downregulation in tubular epithelial cells (TECs) as a hallmark of diabetic kidney disease (DKD), correlating with disease severity.
• SIRT6 epigenetically silences *Nlrp3* transcription by deacetylating H3K9, thereby suppressing NLRP3 inflammasome activation and mitigating tubular injury.
• TEC-specific *Sirt6* deletion exacerbates renal dysfunction in DKD models, whereas its overexpression confers protection, establishing a causal role in disease progression.
• Pharmacological SIRT6 activation attenuates DKD pathology, highlighting its therapeutic potential for tubulointerstitial injury in diabetes.

## Graphical abstract

**Figure:**
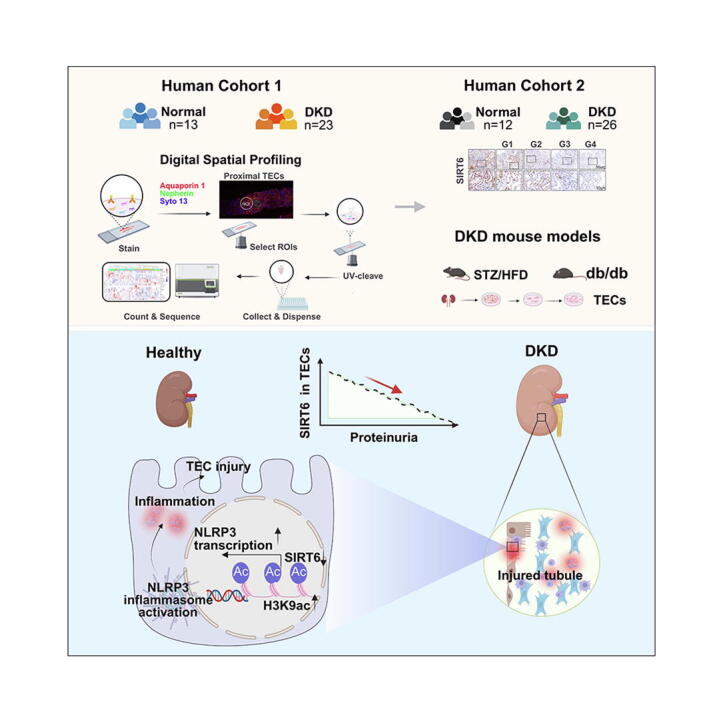

## Introduction

Diabetic kidney disease (DKD), a main microvascular complication of diabetes, is the leading cause of chronic kidney diseases and end-stage kidney disease worldwide [1]. Despite significant advances in glycemic control and blood pressure management, these interventions have only mitigated but not eliminated the risk of DKD progression [2]. This underscores the critical demand for novel curative targets and strategies to overcome the remaining clinical challenges in DKD.

The pathogenesis of DKD is multifaceted and has traditionally been viewed through a glomerulocentric lens, with proteinuria primarily attributed to glomerular abnormalities [3]. However, emerging evidence indicates that proximal tubular epithelial cells (TECs) injury may precede glomerular damage, and that tubulointerstitial pathology is the strongest histopathological predictor of renal functional decline [4]. This paradigm is further supported by clinical trial data showing that angiotensin-converting enzyme (ACE) inhibitors, angiotensin receptor blockers (ARBs), and sodium-glucose cotransporter-2 (SGLT2) inhibitors confer renoprotective effects, at least in part by targeting proximal TECs, thereby highlighting the pivotal role of tubular dysfunction in DKD progression [5,6]. Despite these advances, the molecular mechanisms underlying TEC injury and degeneration in DKD remain poorly defined. A deeper exploration of tubulointerstitial pathobiology is therefore crucial for developing novel therapeutic strategies to combat DKD.

Histone deacetylases (HDACs) are a conserved family of epigenetic enzymes that regulate chromatin structure by removing acetyl groups from histone lysine residues, thereby broadly influencing gene transcription in health and disease. Owing to their well-defined catalytic domains, HDACs have become attractive therapeutic targets, and several inhibitors are currently under evaluation in preclinical and clinical trials, particularly for cancer treatment [7]. However, their therapeutic potential in DKD remains largely unexplored. Among HDACs, SIRT6, an NAD^+^-dependent HDAC within the sirtuin (SIRT) family, exhibits diverse catalytic activities, including deacetylation, diacylation, and mono-ADP-ribosylation [8], which modifies histone H3 at lysine 9 (H3K9) and H3K56, thereby controlling diverse processes such as transcription, DNA repair, and cellular senescence [9,10]. Previous studies have shown that different sirtuin members exert renoprotective effects in DKD. SIRT1 primarily attenuates tubular injury through modulation of hypoxia-induced HIF-1α signaling and suppression of inflammation [11], while SIRT3 preserves mitochondrial integrity and promotes mitophagy [12], thereby maintaining tubular homeostasis under stress conditions [13]. In contrast, evidence for SIRT6 has so far been limited. Although SIRT6 has been implicated in podocyte protection through transcriptional repression of Notch signalling [14], its role in TECs during DKD progression remains largely unexplored. As epigenetic mechanisms are increasingly recognized as pivotal modulators of inflammatory injury in renal tubules, clarifying the specific contribution of SIRT6 offers an opportunity to bridge this mechanistic gap. Such insights may extend beyond molecular understanding toward refining patient risk profiles [15], identifying windows for early intervention, and designing individualized therapeutic approaches in DKD [[15], [16], [17]].

Here, we employed high-resolution digital spatial profiling (DSP) to analyze whole-transcriptome expression in immunostained tubulointerstitial regions of kidney sections from 23 DKD patients and 13 normal controls. We found that *Sirt6* levels in the proximal renal tubular tissue were significantly reduced in DKD patients, showing a negative correlation with 24-hour urinary protein excretion and a positive correlation with estimated glomerular filtration rate (eGFR). This SIRT6 downregulation, accompanied by elevated H3K9 acetylation (H3K9ac) levels, was further validated in an independent patient cohort and replicated in two established DKD mouse models. Functionally, TEC-specific SIRT6 deficiency exacerbated tubular injury and proteinuria in DKD mouse models, whereas SIRT6 overexpression or pharmacological activation effectively attenuated DKD progression. To elucidate downstream mechanisms, we performed RNA-sequencing (RNA-seq) and Cleavage Under Targets and Tagmentation (CUT&Tag) sequencing [18,19], identifying direct targets of SIRT6. Notably, SIRT6 represses *Nlrp3* transcription by modulating H3K9ac, thereby suppressing NLRP3 inflammasome activation and subsequent TEC injury, ultimately attenuating DKD progression. Our findings suggest that targeting renal tubular SIRT6 holds promise as an approach for DKD management.

## Methods

Detailed experimental protocols are available in the Supplementary Materials and Methods. Target gene primers: See Tab. S3. Antibodies: Comprehensive list provided in Tab. S4.

### Human renal tissue

Renal biopsy samples from patients with DKD and histologically normal renal tissue (from tumor nephrectomy margins) were collected from the Department of Nephrology, China-Japan Friendship Hospital under approved protocols (2024-KY-129-1). All control specimens were rigorously screened to exclude pre-existing renal pathology. Comprehensive clinical and biochemical characteristics of both cohorts are presented in Tab. S1, 2. All DKD biopsies were histologically confirmed by certified renal pathologists and classified according to Kidney Disease: Improving Global Outcomes (KDIGO) eGFR-based guidelines [1]. This study adhered to the ethical principles of the Declaration of Helsinki.

### DSP and RNA analysis

Formalin-fixed, paraffin-embedded (FFPE) renal tissue sections (4 µm) were deparaffinized and subjected to RNA target retrieval under pressurized heating in Tris-EDTA buffer (Bio SB, USA). To remove RNA-binding proteins, sections were enzymatically digested (GeoMx DSP RNA Slide Prep Kit, NanoString), followed by post-fixation in 16 % formaldehyde (5 min, RT). For spatial transcriptomic analysis, sections were hybridized overnight with probes targeting 18,000 endogenous transcripts (GeoMx Panel and Seq Code Kit, NanoString). Each probe contained a DNA oligonucleotide with a unique molecular identifier (UMI), RNA-binding sequence, and primer site linked via a UV-cleavable spacer.

For ROI selection, slides were then immunostained with primary antibodies against Nephrin and Aquaporin 1, along with the nuclear marker Syto 13 (NanoString), followed by incubation with fluorescently labeled secondary antibodies at 37 °C for 30 min. ROIs were delineated using the GeoMx Digital Spatial Profiler (NanoString). UV cleavage released oligonucleotides, which were aspirated and processed for Illumina sequencing via adapter ligation and dual-indexing. Libraries were sequenced on an Illumina NovaSeq 6000. This study was conducted in collaboration with Fynn Biotechnologies Ltd. (Shandong, China).

### Animals

All animal experiments were performed in compliance with ARRIVE guidelines 2.0 and approved by the Institutional Animal Care and Use Committee (IACUC) of China-Japan Friendship Hospital (Protocol #ZRDWLL230011). Male littermate mice (C57BL/6J background) were randomly assigned to experimental groups using a blinded design. Animals were maintained under specific pathogen-free (SPF) conditions at a controlled temperature (22–24 °C) with a 12-hour light/dark cycle. Autoclaved cages, bedding, and irradiated food and water are provided ad libitum. Male mice were selected based on their well-documented metabolic susceptibility in prior studies, minimizing potential sex-specific variability.

### Generation of TEC-specific *Sirt6* knockout mice

To generate TEC-specific *Sirt6* knockout mice (*Sirt*6^ΔTEC^), we crossed *Sirt6*^flox/flox^ (*Sirt6*^fl/fl^) (Cyagen, S-CKO-11371) with *Ggt1-Cre* mice (Cyagen, C001028) on a C57BL/6 backgroud. *Sirt6*^fl/fl^ littermates served as controls. For genotyping, tail DNA was analyzed by PCR using: *Sirt6* primers (F: 5′-GTCTTTGTTGTTTCTGAAGGGGTG-3′; R: 5′-AAGATGCAGCTCTACTTGTCTAGG-3′; wide-type: 183 bp; *Sirt6*^fl/fl^: 250 bp; heterozygous: both bands). *Ggt1-Cre* primers (F: 5′-GACGATGAAGCATGTTTAGCTGG-3′; R: 5′-GACGATGAAGCATGTTTAGCTGG-3′; Cre^+^: 428 bp, Cre^-^: no band).

### TECs isolation

TECs were isolated from mouse kidneys using an established protocol with minor modifications [20,21]. Briefly, dissected renal cortices were mechanically minced into 1–2 mm^3^ fragments and subjected to enzymatic digestion with 0.1 % collagenase type II (#17101015, Thermo Fisher Scientific) at 37 °C for 40 min. Digestion was terminated by adding DMEM/F12 complete medium. Tubular fragments were separated from glomeruli and renal mesenchyme through percoll gradient centrifugation (#CP8331, Coolaber). The isolated tubules were washed twice with pre-cooled DMEM/F12 complete medium. Tubular fragments were cultured in DMEM/F12 complete medium for 48 h prior to experiments.

### CUT&Tag

The CUT&Tag assay was performed using the Hyperactive^TM^ In-Situ ChIP Library Prep Kit (#TD901-TD902, Vazyme Biotech) following manufacturer's instructions [18,19]. Briefly, Target-specific chromatin profiling was achieved using an anti-H3K9ac antibody (#A21107, Abclonal) to guide the ChiTag enzyme to H3K9ac-marked genomic regions, where it simultaneously fragmented DNA and incorporated sequencing adapters. The tagged DNA fragments were then released from nuclei and amplified by PCR, with library quality verified using an Agilent 2100 Bioanalyzer before sequencing and downstream analysis (OE Biotech, Shanghai).

### Statistics

Data are expressed as mean mean ± SEM and analyzed using GraphPad Prism 9 (v9.5.1). Normality was assessed using the Kolmogorov-Smirnov test. For normally distributed data, two-group comparisons were performed with unpaired two-tailed Student’s *t*-tests, whereas single- or multi-variable comparisons were conducted using one- or two-way ANOVA followed by Tukey’s multiple comparisons test, respectively. Nonparametric data were analyzed using the Kruskal-Wallis test with Dunn’s post hoc correction. Correlations were assessed via Spearman’s rank correlation analysis.

### Data availability

The multi-omics datasets generated in this study are publicly available in the NCBI Gene Expression Omnibus (GEO): RNA-seq (GSE291755); CUT&Tag (GSE291844); DSP (GSE294519).

## Results

### Proximal tubular SIRT6 expression is reduced in patients and mice with DKD

**Fig. 1:**
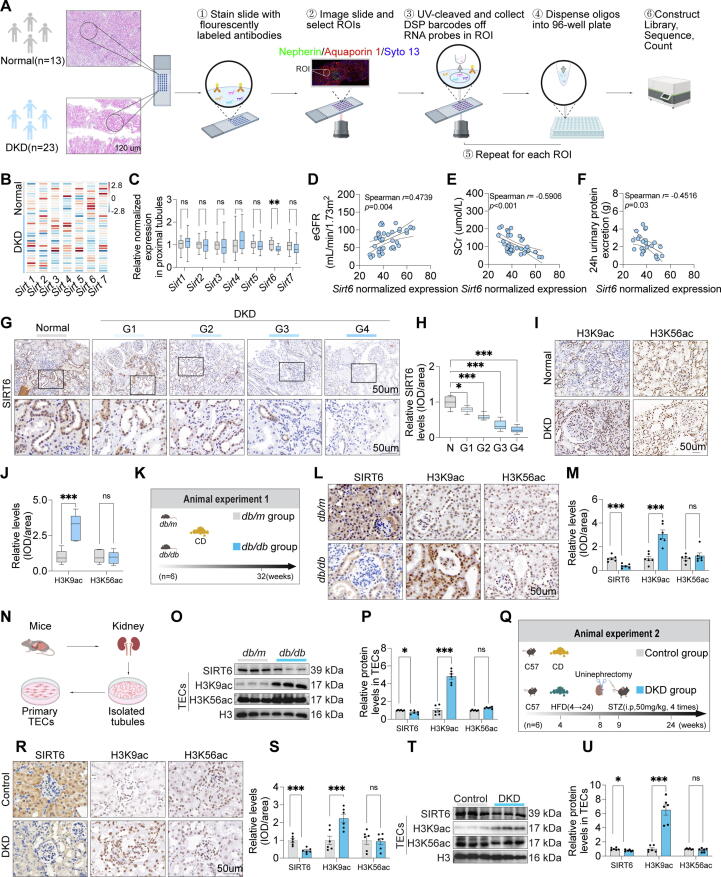
Proximal tubular SIRT6 expression is reduced in patients and mice with DKD. A Schematic overview of digital spatial profiling (DSP) performed on human kidney biopsies. Formalin-fixed paraffin-embedded (FFPE) tissue section from DKD patients (n = 23) and normal controls (n = 13) were stained with H&E to guide region selection. Regions of interest (ROIs) were annotated using immunofluorescence for tubular (Aquaporin 1) and podocyte (Nephrin) markers. Photocleavable oligonucleotide-tagged probes were UV-released and quantified by next-generation sequencing. B, C Heatmap (B) and mRNA levels (C) of SIRT family genes in proximal tubules from DKD and control cohorts. Data normalized to Quartile 3 count. D-F Correlation analyses between proximal tubular *Sirt6* mRNA levels and clinical parameters: estimated glomerular filtration rate (eGFR; D), serum creatinine (SCr; E), and 24-hour urinary protein (F). G, H Representative immunohistochemistry (IHC) and quantification of SIRT6 in human renal cortices stratified by DKD stages (Normal: n = 12; G1-G4: n = 4–10 per group), scale bars, 50 µ m. I, J IHC analysis of H3K9ac and H3K56ac in DKD (n = 26) vs. controls (n = 12), scale bars, 50 µ m. K Experimental design for mouse studies. L-M IHC staining and integrated optical density (IOD) quantification of SIRT6, H3K9ac, and H3K56ac in murine kidneys (n = 6/group). N Isolation protocol for tubular epithelial cells (TECs) from murine kidneys. O-P Protein expression of SIRT6 and histone modifications in isolated TECs was analyzed by Western blot with densitometry (n = 6/group). Q High-fat diet and streptozotocin (HFD/STZ)-induced DKD model timeline. R-S IHC staining and integrated optical density (IOD) quantification of SIRT6, H3K9ac, and H3K56ac in murine kidneys (n = 6/group), scale bars, 50 µ m. T-U Protein expression of SIRT6 and associated histone modifications in isolated TECs were analyzed by Western blot followed by densitometric analysis (n = 6/group). Data are mean ± SEM. Two-way ANOVA with Tukey’s multiple comparisons test (C, J, M, P, S, U); One-way ANOVA with Tukey’s post hoc analysis (H); Spearman’s correlation (D-F). *p < 0.05, ^**^p < 0.01, ^***^p < 0.001.

We initially evaluated the expression profiles of *SIRTs* in tubulointerstitial regions of human renal biopsies using DSP, a technology that enables spatially resolved quantification of gene expression within specific areas of tissue sections[22] (Fig. 1A). Our analysis demonstrated significantly reduced *Sirt6* mRNA levels in the proximal renal tubular tissue of DKD patients (n = 23) compared to normal controls (n = 13) (Fig. 1B, C). Notably, *Sirt6* mRNA expression in proximal renal tubules exhibited a positive correlation with eGFR, while demonstrating inverse associations with both serum creatinine (SCr) levels, 24-hour urinary protein excretion and HbA1c (Fig. 1D-F; Fig. S1A-D). Other clinical parameters, including additional diabetes-related variables, are provided in Tab. S1.

To validate these observations at the protein level, we performed immunohistochemistry (IHC) in an independent cohort comprising 12 normal individuals and 26 DKD patients. SIRT6 protein levels were markedly lower in proximal TECs of DKD patients than in normal controls and inversely correlated with disease severity (Fig. 1G, H). Consistent with its role as a histone deacetylase, H3K9ac levels were elevated in TECs from DKD patients, while H3K56ac levels remained unchanged (Fig. 1I, J) (Tab. S2). These findings suggest that proximal tubular SIRT6 and its associated histone modification, H3K9ac, may provide mechanistic insights into human DKD progression.

We further examined SIRT6 expression in kidney tissues and isolated TECs from two established DKD mouse models [20]: *db/db* mice and uninephrectomized mice treated with streptozotocin (STZ) and high-fat diet (HFD) [23]. Mirroring clinical observations, SIRT6 expression was markedly decreased in TECs from both models, accompanied by elevated H3K9ac levels and renal expression of kidney injury molecule-1 (KIM-1), a recognized marker of tubular injury[4] (Fig. 1K-U; Fig. S1E-P). Furthermore, SIRT6 levels in TECs negatively correlated with DKD severity (Fig. S1Q-V). Taken together, these findings demonstrate that reduced tubular SIRT6 expression is a common feature in both human and murine DKD, implicating the SIRT6-H3K9ac regulatory axis in tubular injury and disease progression.

### TEC-specific SIRT6 deficiency exacerbates renal injury in DKD mice

**Fig. 2:**
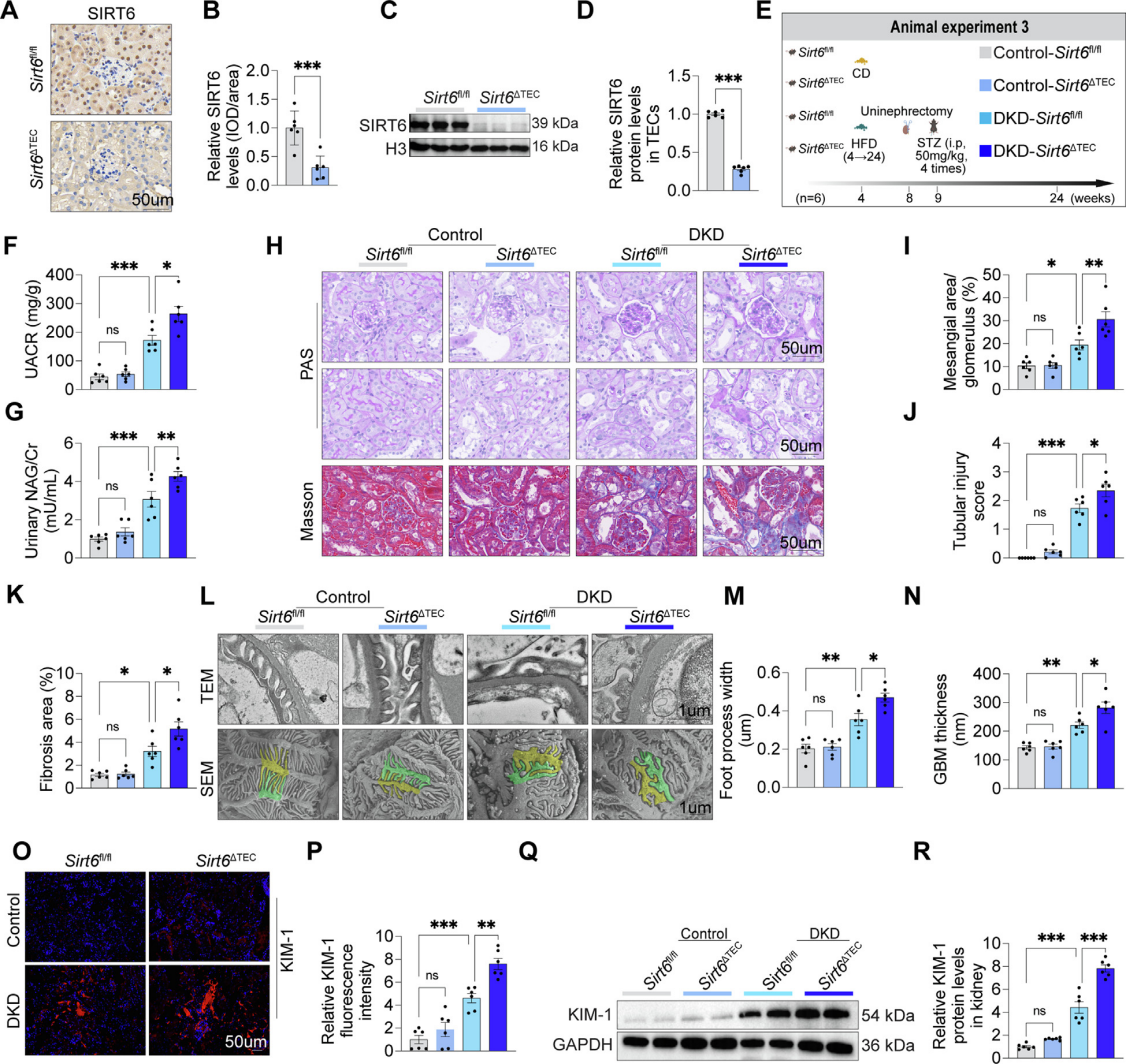
TEC-specific SIRT6 deficiency exacerbates renal injury in DKD mice. A, B IHC of SIRT6 expression in renal tubules from two groups of mice, with corresponding quantitation (n = 6/group), scale bar, 50 µ m. C, D Western blot analysis of SIRT6 protein levels in isolated TECs (n = 6/group). E Schematic overview of the experimental design, outlining dietary and genetic interventions. F, G UACR and urinary N-acetyl-β-D-glucosaminidase-to-creatinine ratio (NAG/Cr) levels in four groups of mice (n = 6/group). H-K Renal pathology assessment showing periodic acid-Schiff (PAS) staining (first, second row), Masson's trichrome staining (third row), and quantitative analyses of (I) mesangial area/glomerulus, (J) tubular injury score, and (K) fibrosis area (n = 6/group), scale bars, 50 µ m. (L-N) Transmission electron microscopy (TEM) and scanning electron microscope (SEM) images (scale bars, 1 um) and quantitative measurements of (M) foot process width and (N) glomerular basement membrane (GBM) thickness (n = 6/group), scale bars, 1 µm. O, P Kidney injury molecule-1 (KIM-1) expression was visualized by immunofluorescence (red) with DAPI counterstain (blue) and quantified (n = 6/group), scale bar, 50 µ m. Q, R Western blot analysis confirmed KIM-1 protein levels in renal tissues (n = 6/group). Data represent mean ± SEM. Unpaired *t*-test (B, D); Two-way ANOVA with Tukey's multiple comparisons (F, G, J, M, N, P, R); Kruskal-Wallis test with Dunn's post hoc analysis (I, K). Significance levels: *p < 0.05, ^**^p < 0.01, ^***^p < 0.001. (For interpretation of the references to colour in this figure legend, the reader is referred to the web version of this article.)

To elucidate the functional contribution of SIRT6 in TECs to DKD progression, we established a novel genetic model through conditional ablation of *Sirt6* specifically in renal tubules, designated *Ggt1*-Cre; *Sirt6*^fl/fl^ (*Sirt*6^ΔTEC^). Successful knockout of *Sirt6* was confirmed by tail genotyping, IHC analysis of kidney sections, and western blot analysis of isolated TECs (Fig. 2A–D; Fig. S2A). DKD was induced in both *Sirt6*^fl/fl^ and *Sirt*6^ΔTEC^ mice through HFD administration followed by uninephrectomy and STZ injection, while control groups for both strains underwent sham operations and were maintained on a normal chow diet (Fig. 2E). Compared to *Sirt6*^fl/fl^ controls, diabetic *Sirt*6^ΔTEC^ mice exhibited elevated urine albumin-to-creatinine ratio (UACR) [24], and urinary N-acetyl-β-D-glucosaminidase-to-creatinine ratio (NAG/Cr) levels [25] (Fig. 2F, G), along with more severe glomerular [26], and podocyte injuries [24], as assessed by morphological analyses (Fig. 2H-N; Fig. S2B, C). Additionally, TEC-specific SIRT6 deficiency significantly exacerbated renal tubular injury [27], as evidenced by increased expression of KIM-1 in the kidney (Fig. 2O-R). Notably, levels of H3K9ac, but not H3K56ac, were substantially elevated in isolated TECs from diabetic mice, with a more pronounced increase observed in *Sirt*6^ΔTEC^ mice. IHC staining further validated the increased H3K9ac levels in TECs from *Sirt*6^ΔTEC^ DKD mice (Fig. S2D-G). Taken together, these results establish SIRT6 as a crucial renoprotective factor that attenuates tubular epithelial injury and slows DKD progression.

### TEC-specific SIRT6 deficiency promotes renal tubular inflammation and fibrogenesis

**Fig. 3:**
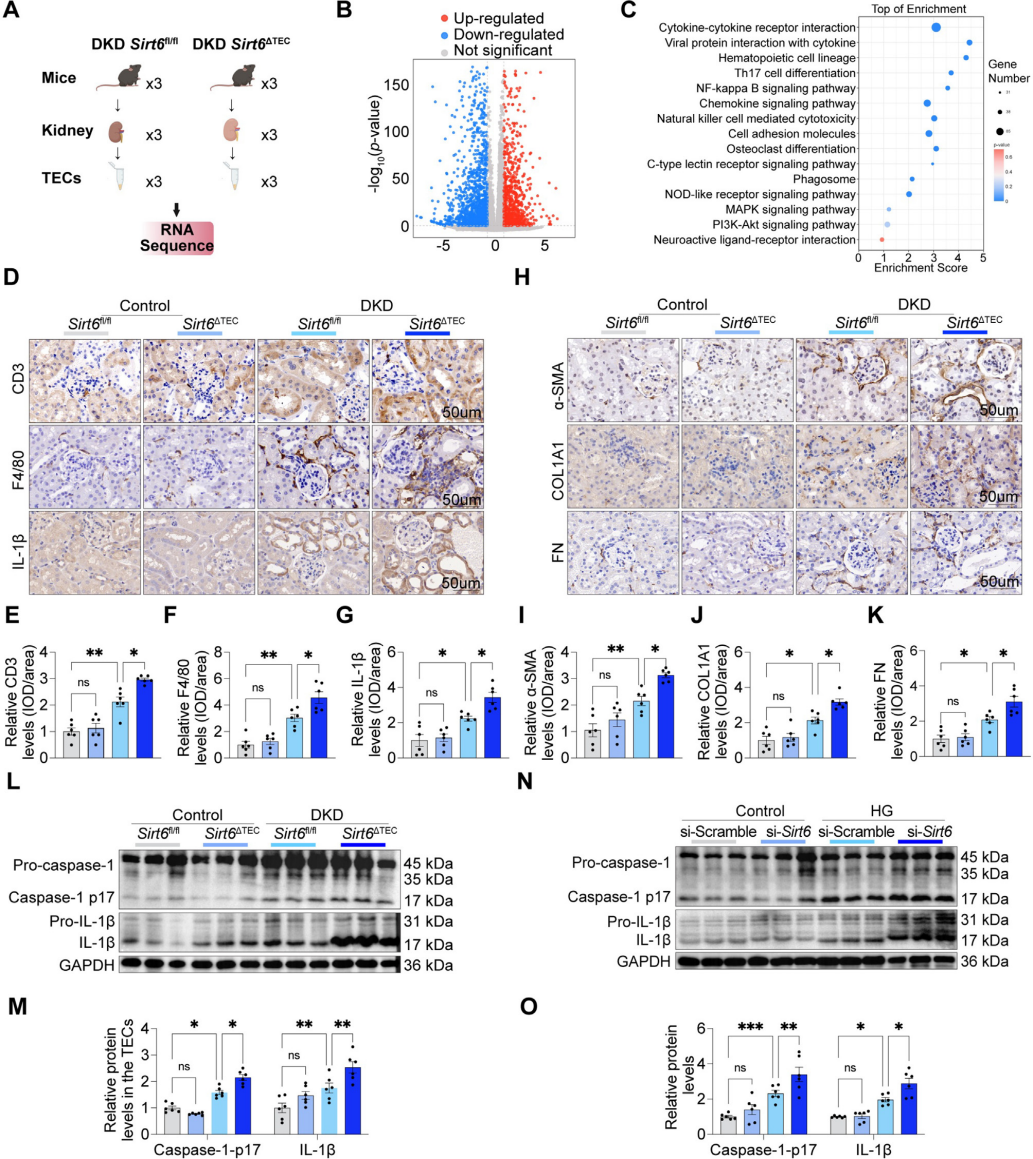
TEC-specific SIRT6 deficiency promotes renal tubular inflammation and fibrogenesis. A Experimental workflow for identifying SIRT6-regulated pathways in DKD. B Volcano plot of differentially expressed genes in TECs from *Sirt6*^fl/fl^ and *Sirt*6^ΔTEC^ DKD mice (red: upregulated; blue: downregulated; threshold: |log_2_FC| > 2, *P* < 0.05). C KEGG pathway analysis of RNA-seq data from TECs. D-K Representative (D, H) IHC and quantitative analysis of (E) CD3, (F) F4/80, (G) IL-1β, (I) α-SMA, (J) COL1A1, and (K) fibronectin (FN) expression in renal tissues (n = 6/group), scale bars, 50 µ m. L, M Western blot analysis demonstrated significant elevation of cleaved Caspase-1 and mature IL-1β in isolated TECs from four groups (n = 6/group). N, O Similarly, high glucose (HG) treatment induced cleavage of Caspase-1 and mature IL-1β in HK-2 cells (n = 6/group). All densitometry data represent mean ± SEM. Two-way ANOVA with Tukey's multiple comparisons test (E, F, G, I, J, K, M, O). *p < 0.05, ^**^p < 0.01, ^***^p < 0.001. (For interpretation of the references to colour in this figure legend, the reader is referred to the web version of this article.)

To explore the molecular mechanisms underlying the exacerbated tubular injury observed in *Sirt*6^ΔTEC^ mice, we conducted transcriptomic profiling of TECs isolated from *Sirt6*^fl/fl^ and *Sirt*6^ΔTEC^ DKD mice (Fig. 3A). KEGG pathway enrichment analysis revealed a pronounced activation of pro-inflammatory signaling cascades with TEC-specific SIRT6 deficiency (Fig. 3B, C). These transcriptomic results aligned well with our IHC observations, which showed increased inflammation after *Sirt6* knockout (Fig. 3D–G). Given that inflammation is a key driver of renal injury and fibrogenesis[28], these results further support a protective role of SIRT6 (Fig. 3H–K; Fig. SA-D). Among the enriched inflammatory pathways, the NOD-like receptor signaling pathway is prominently represented. While NLRP3 upregulation in podocytes has previously been implicated in DKD progression [29], a similar mechanism appears to operate in TECs, in this context, NLRP3 inflammasome activation and assembly drives IL-1β maturation are known to promote both tubular injury and fibrosis [4]. Consistently, RNA-Seq enrichment analysis identified alteration of the IL-1β production pathway, suggesting that TEC-specific SIRT6 deficiency triggers NLRP3 inflammasome–mediated inflammatory responses (Fig. 3L, M; Fig. S3E). This was further supported by elevated levels of downstream inflammatory mediators in *Sirt*6^ΔTEC^ DKD mice.

To validate these findings *in vitro*, we performed siRNA-mediated silencing of *Sirt6* in HK-2 cells, demonstrating successful knockdown through mRNA and protein analyses (Fig. S3F-H). Under high glucose (HG) conditions, SIRT6 deficiency further enhanced the levels of IL-1β and cleaved caspase-1 (p17), indicating enhanced inflammasome activation (Fig. 3N, O). These molecular changes were accompanied by markers of tubular cell injury, supporting the pro-inflammatory and injurious consequences of SIRT6 loss in a diabetic environment. Collectively, these results highlight inflammation as a major downstream consequence of SIRT6 deficiency in DKD, potentially mediated via the NLRP3 pathway.

### SIRT6 suppresses NLRP3-mediated inflammation through H3K9 deacetylation

**Fig. 4:**
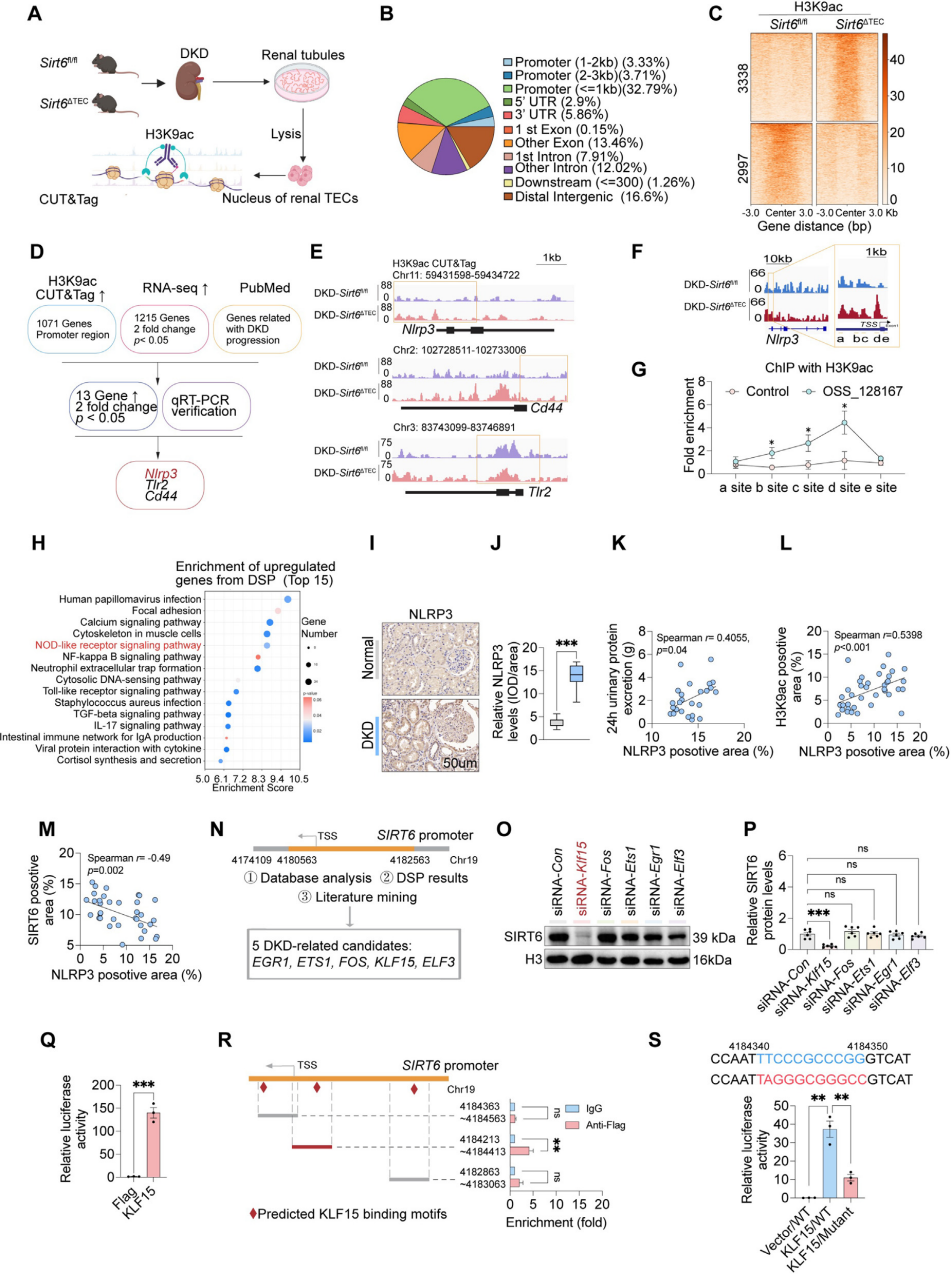
SIRT6 suppresses NLRP3-mediated inflammation through H3K9 deacetylation. A Schematic of CUT&Tag workflow for histone modification profiling. B Genome-wide distribution of H3K9ac enrichment in TECs from *Sirt6*^fl/fl^ and *Sirt*6^ΔTEC^ DKD mice, showing predominant promoter localization. C Heatmaps and quantitative analysis of differential H3K9ac peaks between genotypes (top: increased peaks; bottom: decreased peaks in *Sirt*6^ΔTEC^). D Bioinformatics pipeline for identifying SIRT6 downstream targets. E Browser tracks of H3K9ac enrichment at promoter regions of *Nlrp3*, *Tlr2* and *Cd44*. F Schematic of *Nlrp3* promoter showing primer locations (sites a-e) and H3K9ac peaks (sites b-d). G ChIP-qPCR analysis of H3K9ac occupancy at *Nlrp3* promoter in HK-2 cells treated with SIRT6 inhibitor OSS_128167 (n = 6/group). H KEGG pathway analysis of upregulated genes identified from proximal renal tubular regions using DSP in DKD patients (n = 23) versus normal controls (n = 13). I, J IHC analysis of NLRP3 expression in renal tissues from patients with DKD (n = 26) and normal controls (n = 12), scale bar, 50 µ m. K-M Correlation analyses between NLRP3 and (K) proteinuria, (L) H3K9ac and (M) SIRT6 in human samples. N Prediction of SIRT6 promoter-binding transcription factors (TFs). Bioinformatic screening, DSP results and literature review identified potential TFs binding to the SIRT6 promoter. TSS, transcription start site. O-P SIRT6 protein levels in HK-2 cells transfected with siRNA for 48 h (normalized to controls). Q Luciferase reporter assay in HK-2 cells transfected with KLF15-overexpressing or control plasmids, followed by HG treatment for 24 h. Data show promoter activity driven by the SIRT6 promoter fragment. R ChIP-qPCR analysis of Flag-KLF15 enrichment at the SIRT6 promoter in HK-2 cells (anti-Flag vs. IgG control). Regions scanned are indicated. S Luciferase activity of wild-type (WT) or mutant SIRT6 promoter constructs in HK-2 cells overexpressing KLF15 (HG for 24 h). Blue: KLF15 motif; red: mutated sequence. Data represent mean ± SEM. Unpaired *t*-test (G, J, Q, R); Two-way ANOVA with Tukey's multiple comparisons test (P, S); Spearman’s correlations (K, L, M). *p < 0.05, ^**^p < 0.01, ^***^p < 0.001. (For interpretation of the references to colour in this figure legend, the reader is referred to the web version of this article.)

To determine whether SIRT6 modulates inflammation through epigenetic mechanisms, we performed genome-wide CUT&Tag analysis to profile H3K9ac-regulated genes in TECs (Fig. 4A). Total TECs were isolated from *Sirt6*^fl/fl^ and *Sirt*6^ΔTEC^ DKD mice, followed by CUT&Tag profiling using an anti-H3K9ac antibody. Subsequent deepTools analysis disclosed a marked enrichment of H3K9ac peaks in TECs from *Sirt*6^ΔTEC^ mice compared to *Sirt6*^fl/fl^ controls. These peaks were predominantly located in promoter and upstream regulatory regions of genes, indicating that SIRT6 may suppress transcription by limiting H3K9ac accumulation in these loci (Fig. 4B; Fig. S4A).

Comparative profiling identified 14,058 genes with increased H3K9ac binding in *Sirt*6^ΔTEC^ mice as compared to *Sirt6*^fl/fl^ controls, including 3,338 genes with significantly elevated peaks (Log_2_ fold change (FC) > 0.8, *p* < 0.05), while only 149 genes showed no significant change (Fig. 4C). Integration of RNA-seq data (FC > 2, *p* < 0.05) and CUT&Tag results (promoter region), combined with PubMed database mining, identified *Nlrp3*, *Tlr2*, and *Cd44* as candidate targets. H3K9ac enrichment at their promoter regions was visualized, and qPCR validation confirmed significant *Nlrp3* upregulation upon *Sirt6* deletion. These results, consistent with IHC staining, support a direct transcriptional repression of *Nlrp3* by SIRT6 (Fig. 4D, E; Fig. S4B-D).

Chromatin immunoprecipitation (ChIP) assays using primers targeting the *Nlrp3* promoter further confirmed H3K9ac enrichment in both mouse TECs and HK-2 cells (Fig. 4F; Fig. S4E-I). Pharmacological inhibition of SIRT6 using OSS_128167 enhanced H3K9ac levels and its recruitment to the *Nlrp3* promoter (Fig. 4G; Fig. S4J, K). To delineate the functional consequences of SIRT6 suppression under HG conditions, we quantified key inflammasome components via immunoblotting, including NLRP3, cleaved Caspase-1 (p17), IL-1β, and Fibronectin (FN). HG exposure increased the expression of all four proteins, which was further exacerbated by SIRT6 inhibition but attenuated by MCC950, a selective NLRP3 inhibitor [30] (Fig. S4L-P).

Consistent with these experimental results, KEGG analysis of upregulated genes from proximal renal tubular DSP data comparing DKD patients and normal controls revealed significant enrichment of the NOD-like receptor pathway (Fig. 4H). In renal biopsies from DKD patients, NLRP3 expression was markedly elevated in TECs, and positively correlated with both 24-hour urinary protein excretion and H3K9ac levels, while inversely correlated with SIRT6 expression (Fig. 4I–M). These findings collectively support the existence of a functional SIRT6–H3K9ac–NLRP3 axis that contributes to renal inflammation and disease progression in DKD.

To elucidate the upstream regulatory mechanisms controlling SIRT6 transcription in tubular cells, we performed integrative analyses across multiple transcription factor prediction databases. Candidate regulators were cross-referenced with differentially expressed genes in DKD from our DSP dataset, and combined with literature screening, five transcription factors, EGR1, ETS1, KLF15, FOS, and ELF3, were predicted to bind the SIRT6 promoter (Fig. 4N).

Functional screening via siRNA-mediated knockdown demonstrated that KLF15 depletion significantly reduced SIRT6 mRNA and protein levels, as confirmed by qPCR and Western blot analyses (Fig. 4O, P; Fig. S4Q). Furthermore, luciferase reporter assays established that KLF15 directly activates SIRT6 promoter-driven transcription, supporting its role as a transcriptional regulator (Fig. 4Q).

To define the molecular basis of this regulation, we analyzed the SIRT6 promoter using the JASPAR database, identifying three conserved structural domains. ChIP assays confirmed specific enrichment of KLF15 at the *Sirt6* promoter locus (chr19: 4,184,213–4,184,413 bp) (Fig. 4R). Importantly, Targeted mutagenesis of the core KLF15-binding site (chr19: 4,184,340–4,184,350 bp) abolished *Sirt6* promoter activation in luciferase assays, demonstrating that this *cis*-regulatory element is essential for KLF15-mediated transcriptional regulation (Fig. 4S).

### The protective effects of SIRT6 against DKD rely on suppression of NLRP3 inflammasome activation

**Fig. 5:**
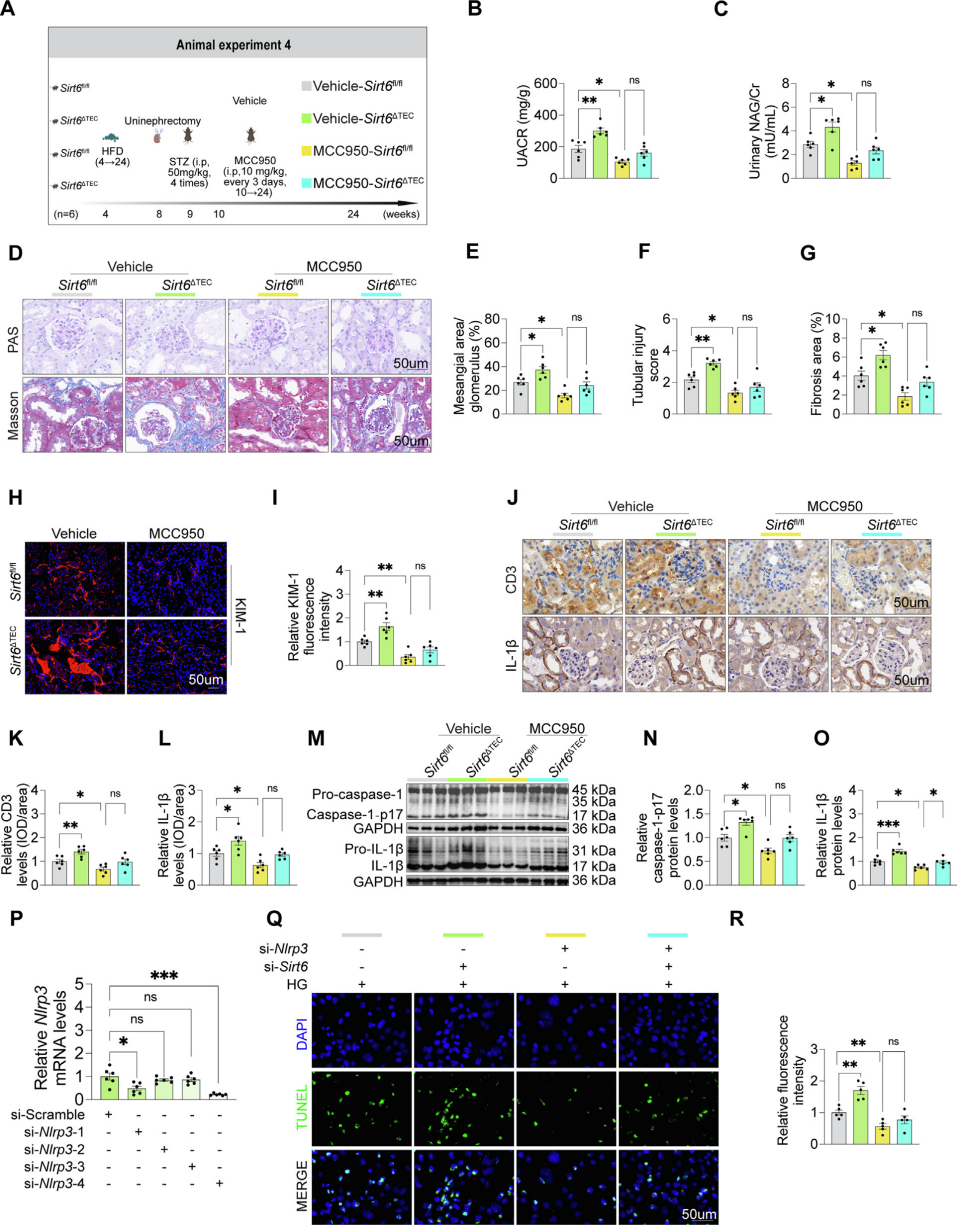
The protective effects of SIRT6 against DKD rely on suppression of NLRP3 inflammasome activation. A Experimental design schematic. B, C UACR and urinary NAG/Cr levels in four groups of mice. (n = 6/group). D-G Renal histopathological assessment showing PAS staining (first row) and Masson's trichrome staining (second row) with quantitative analyses of (E) mesangial area/glomerulus, (F) tubular injury score, and (G) fibrotic area (n = 6/group), scale bar, 50 µ m. H, I Immunofluorescence staining of KIM-1 (red) with DAPI nuclear counterstain (blue) and quantification (n = 6/group), scale bar, 50 µ m. J-L IHC of CD3 (first row) and IL-1β (second row) expression with quantification in renal tissues (n = 6/group), scale bar, 50 µ m. M-O Western blot revealed significant increases in cleaved Caspase-1 and mature IIL-1β in isolated TECs across experimental conditions (n = 6/group). P Relative *Nlrp3* mRNA levels in HK-2 cells under different treatment conditions (n = 6/group). Q-R Immunofluorescence staining of TUNEL (green) with DAPI nuclear counterstain (blue) and quantification (n = 6/group), scale bar, 50 µ m. Data represent mean ± SEM. One-way ANOVA with Tukey's post hoc analysis (P); Two-way ANOVA with Tukey's multiple comparisons test (B, C, F, I, K, L, N, O, R); Kruskal-Wallis test with Dunn's multiple comparisons correction (E, G). Statistical significance: *p < 0.05, ^**^p < 0.01, ^***^p < 0.001. (For interpretation of the references to colour in this figure legend, the reader is referred to the web version of this article.)

To further validate the proposed mechanism *in vivo*, we administered the selective NLRP3 inhibitor MCC950 [30,31] to both *Sirt6*^fl/fl^ and *Sirt*6^ΔTEC^ DKD mice (Fig. 5A). As anticipated, MCC950 significantly attenuated STZ/HFD-induced increases in UACR, and urinary NAG/Cr levels (Fig. 5B, C). Furthermore, MCC950 effectively mitigated TEC-specific SIRT6 deficiency-induced renal pathological features, including glomerular injury, interstitial fibrosis, and tubular injury in DKD mice (Fig. 5D–I). Importantly, MCC950 treatment also abolished NLRP3 inflammasome activation and its downstream inflammatory cascades in *Sirt*6^ΔTEC^ mice (Fig. 5J–O). Consistently, in HG-treated HK-2 cells, SIRT6 knockdown significantly increased the proportion of TUNEL-positive cells, whereas concomitant NLRP3 knockdown markedly attenuated these changes (Fig. 5P–R). Together, these *in vivo* and *in vitro* findings demonstrate that the protective effects of SIRT6 in DKD are mediated, at least in part, through suppression of NLRP3-mediated inflammation.

### SIRT6 overexpression or activation ameliorates renal inflammation and injury in DKD

**Fig. 6:**
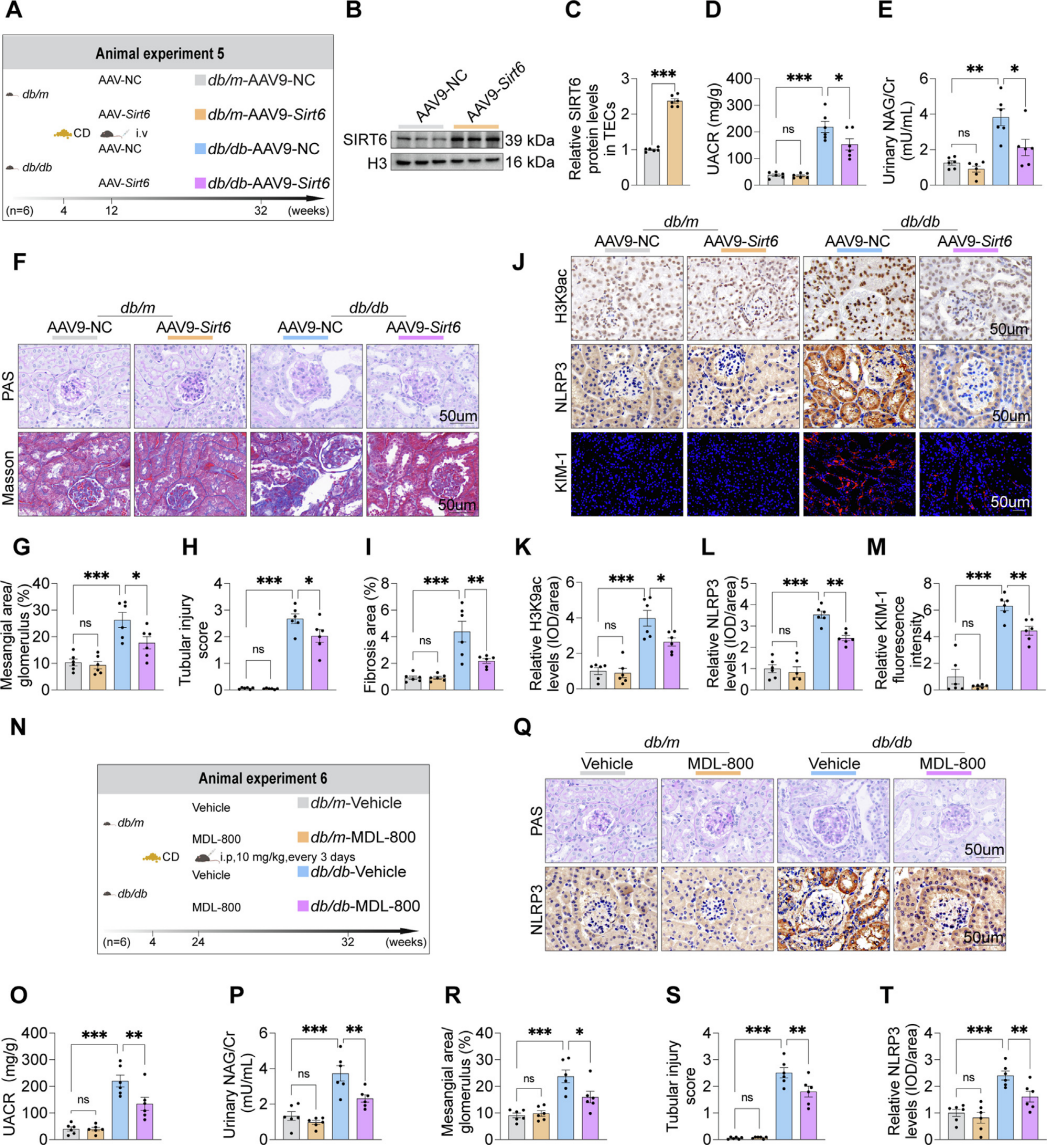
SIRT6 overexpression or pharmacological activation ameliorates renal inflammation and injury in DKD. A Experimental design schematic for SIRT6 overexpression. B, C Western blot of SIRT6 in isolated TECs (n = 6/group). D, E UACR and urinary NAG/Cr levels in four groups of mice (n = 6/group). F-I Renal histopathologic assessment by PAS staining (first row) and Masson's trichrome staining (second row) with quantification of (G) mesangial area, (H) tubular injury score, and (I) fibrosis area (n = 6/group), scale bars, 50 µm. J-M IHC of H3K9ac (first row) and NLRP3 (second row), and immunofluorescence staining of KIM-1 (third row, red) with DAPI counterstain (blue), along with quantitative analyses (n = 6/group), scale bars, 50 µ m. N Experimental design schematic for SIRT6 activation. O, P UACR and urinary NAG/Cr levels in four groups of mice (n = 6/group). Q-T Renal pathology evaluation showing PAS staining (first row) and IHC for NLRP3 (second row), with quantification (n = 6/group), scale bar, 50 µ m. Data represent mean ± SEM. Unpaired *t*-test (C). Two-way ANOVA with Tukey's multiple comparisons test (D, E, H, K, L, M, O, P, S, T); Kruskal-Wallis test with Dunn's multiple comparisons correction (G, I, R). Statistical significance: *p < 0.05, ^**^p < 0.01, ^***^p < 0.001. (For interpretation of the references to colour in this figure legend, the reader is referred to the web version of this article.)

To appraise the therapeutic potential of SIRT6 in DKD, we delivered adeno-associated virus (AAV) vectors encoding *Sirt6* under a TEC-specific promoter (AAV9-*Sirt6*) into *db/m* and *db/db* mice (Fig. 6A). Successful *in vivo* gene transfer was confirmed by IHC and western blot analyses, demonstrating elevated SIRT6 expression in renal tubular tissues and isolated TECs (Fig. 6B, C; Fig. S5A-D). AAV9-*Sirt6* administration ameliorated the increases in UACR, and urinary NAG/Cr levels (Fig. 6D, E), and improved renal structure and function in *db/db* mice (Fig. 6F-I). SIRT6 overexpression also attenuated renal inflammation and fibrosis, and reduced tubular injury, as indicated by decreased KIM-1 expression (Fig. 6J-M; Fig. S5E-H). To explore translational relevance, we further treated *db/db* mice with MDL-800, a selective pharmacological SIRT6 activator (Fig. 6N), which recapitulated these protective effects observed with AAV9-*Sirt6* treatment (Fig. 6O-T; Fig. S5I-M). Together, these findings demonstrate that both genetic overexpression and pharmacological activation of SIRT6 effectively mitigate renal tubular inflammation and injury in DKD, highlighting its therapeutic potential.

## Discussion

DKD is a major diabetic complication primarily managed by aggressive intensive glycemic control, blood pressure management, combined with ACE inhibitors or ARBs targeting the renin-angiotensin system. However, these therapies predominantly target glomerular function and often insufficient to halt or reverse disease progression in most patients [32]. Recent therapeutic advances, including the clinical success of SGLT2 inhibitors, non-steroidal mineralocorticoid receptor antagonists [33], and GLP-1 receptor agonists [34], have shifted the paradigm toward recognizing the multifactorial nature of DKD. In particular, the efficacy of SGLT2 inhibitors highlights the critical contribution of tubular dysfunction to disease progression, suggesting that targeting tubular-specific pathways may complement traditional glomerular-centric strategies [35].

Dysregulation of classical signaling pathways in renal cells during diabetes contributes significantly to pathological progression in DKD. Such dysregulation stems not only from genetic variations but also from dynamic epigenetic modifications, including alterations in histone modification, DNA methylation, and noncoding RNA expression [36]. Notably, these epigenetic modifications can perpetuate “metabolic memory” by maintaining diabetes-induced transcriptional programs long after initial glycemic exposure. Early detection of such epigenetic signatures in DKD pathogenesis may enable timely risk stratification and targeted interventions to avert end-stage renal disease progression [37]. In this study, we identify the histone deacetylase SIRT6 as a key epigenetic regulator that attenuates renal TEC injury by inhibiting NLRP3 inflammasome activation, thereby protecting against DKD progression. Our findings unveil a previously unrecognized epigenetic axis in DKD pathogenesis and position SIRT6 as a promising therapeutic target to disrupt the metabolic memory fueling renal decline. Beyond mechanistic relevance, tubular SIRT6 emerges as a candidate biomarker enabling risk prediction, stratification, and early-stage intervention [38]. From a translational standpoint, integration with digital health tools and AI-driven analytics could facilitate proactive, patient-tailored care, representing a step toward predictive, preventive, and personalized medicine [39,40].

Emerging evidence underscores the pivotal role of HDAC-mediated epigenetic regulation in the pathogenesis of kidney diseases [41]. The HDAC family consists of 18 members, which are categorized into four classes (I-IV) that orchestrate chromatin remodeling by counterbalancing histone acetyltransferase activity. Among them, class III HDACs, also referred to as sirtuins (SIRT1-7), are NAD^+^-dependent deacetylases that function as metabolic sensors, linking cellular energy status to oxidative homeostasis [42]. Sirtuins contribute to renal pathogenesis by regulating key processes such as mitochondrial function, inflammation, and autophagy, and their dysregulation has been linked to DKD progression [8]. SIRT1 exerts protective effects not only in TECs [43] but also in mesangial cells [44] and podocytes [45], through antioxidant defenses and enhanced autophagic flux. SIRT3 preserves tubular homeostasis by maintaining mitochondrial integrity [13]. In this study, we identify SIRT6 as a particularly relevant member, conferring protection through epigenetic modulation of inflammatory responses in TECs. Importantly, pharmacological activation of the NAD^+^-SIRT1 axis has already shown translational promise, suggesting that targeting SIRT6 may represent an even more attractive therapeutic avenue in DKD [46].

Transcriptomic technologies, such as bulk RNA-Seq, single-cell or single-nucleus RNA-Seq, have greatly advanced our understanding of renal cellular heterogeneity. However, their inability to preserve spatial information limits their utility in studying region-specific disease mechanisms [47]. This limitation is particularly critical in DKD, where injury exhibits significant regional heterogeneity; for instance, glomerular damage can be exacerbated by inflammatory factors released from adjacent fibrotic tubules, independent of intrinsic glomerular injury. To address this issue, we employed an approach by integrating multiple renal biopsy samples onto a single slice for DSP analysis [48]. This innovative approach not only enhances experimental efficiency but also retains spatial resolution and improving cross-sample comparability. Using this platform, we identified a significant *Sirt6* mRNA downregulation in TECs from 23 DKD patients compared to 13 normal controls. Notably, *Sirt6* expression was inversely correlated with HbA1c, suggesting that poor glycemic control may contribute to its reduction. Moreover, *Sirt6* expression negatively correlated with 24-hour urine protein excretion and positively correlated with eGFR. These findings were further validated by IHC in an independent cohort, which revealed a progressive decline in SIRT6 protein levels with increasing DKD severity.

SIRT6, a key regulator of histone modifications [49], modulates gene transcription through context-dependent deacetylation of H3K9 and H3K56. Previous studies have shown that SIRT6 exerts renoprotective effects through multiple mechanisms, including regulation of autophagy [14], vascular homeostasis [50,51], and fibrotic gene expression [52]. In this study, systematic profiling of SIRT6-associated histone modifications revealed a selective dysregulation of H3K9ac, but not H3K56ac, during DKD progression, implicating SIRT6 as a key epigenetic regulator in disease pathogenesis through H3K9ac-mediated chromatin remodeling. While global *Sirt6* knockout leads to severe systemic abnormalities and early mortality, our TEC-specific knockout model provided critical insights into its renal function. TEC-specific SIRT6 ablation exacerbated STZ/HFD-induced DKD, whereas SIRT6 overexpression or pharmacological activation attenuated disease progression. While other HDACs, such as HDAC5 and HDAC9, have been implicated in tubular injury and fibrosis, their alterations appear variable and context-dependent [53]. However, our DSP analysis of DKD patient biopsies revealed that SIRT6 was the only member consistently downregulated in TECs, whereas other enzymes did not display significant alterations. This selective change highlights SIRT6 as the predominant contributor to H3K9ac dysregulation in TECs during DKD progression, supporting a nonredundant role for SIRT6 in disease pathogenesis.

To identify transcriptional changes downstream of SIRT6 loss during DKD, we performed RNA-Seq on primary renal TECs isolated from *Sirt6*^fl/fl^ and *Sirt*6^ΔTEC^ DKD mice. Our transcriptomic analysis revealed a pronounced enrichment of inflammatory pathways, including the NOD-like receptor signaling pathway, and cytokine-cytokine receptor interactions—key mechanisms implicated in diabetic kidney injury. Although primary TECs provide a more physiologically relevant model than immortalized cell lines, their limited availability has long limited genome-wide epigenetic studies [54]. To address this, we employed CUT&Tag, a high-sensitivity technique that requires only thousands of cells, enabling comprehensive mapping of H3K9ac landscapes in primary TECs from *Sirt6*^fl/fl^ and *Sirt*6^ΔTEC^ DKD mice [55]. This approach not only elucidates the epigenetic regulatory role of SIRT6 in DKD but also establishes a framework for future primary cell-based epigenomic study in kidney disease research.

Using CUT&Tag analysis, we demonstrated that SIRT6 mediated the deacetylation of H3K9 at the *Nlrp3* promoter, thereby suppressing its transcriptional activation. This finding reveals a novel epigenetic mechanism by which SIRT6 regulates NLRP3 inflammasome activity. The NLRP3 inflammasome, a key component of innate immunity, is activated by diverse stimuli, including microbial components, endogenous danger signals, and environmental factors [56]. Tight regulation of NLRP3 is critical to prevent aberrant inflammatory responses, and emerging studies highlight the importance of post-translational modifications, particularly acetylation, in modulating its activity [57]. Previous work has shown that SIRT2 deacetylates NLRP3 at lysine 21/22 (but not K24) in macrophages, with acetylation promoting inflammasome activation [58]. However, conflicting evidence suggests that lysine 24 can also be acetylated, with KAT5 identified as the responsible acetyltransferase both *in vitro* and *in vivo* [59]. These discrepancies may reflect cell type-specific regulation or context-dependent modifications, underscoring the complexity of NLRP3 acetylation in inflammasome control. Our study extends this paradigm by identifying SIRT6 as a critical epigenetic regulator of *Nlrp3* through H3K9 deacetylation at its promoter. This mechanism adds another layer of regulation, linking metabolic sensing (via SIRT6) to inflammasome activity through histone modification.

In addition to genetic evidence, we validated our findings pharmacologically using MDL-800, a highly selective allosteric activator of SIRT6 [60]. Consistent with its reported protective roles in osteoarthritis and diabetic heart failure with preserved ejection fraction [9,61], MDL-800 enhanced SIRT6 activity, reduced H3K9ac enrichment at the NLRP3 promoter, and mitigated tubular injury under diabetic conditions. These data further highlight the therapeutic relevance of targeting SIRT6 in DKD. By contrast, MCC950, an NLRP3 inhibitor that blocks inflammasome assembly [62], showed efficacy in our experiments but was discontinued clinically due to hepatotoxicity [63]. Thus, pharmacological activation of SIRT6 may provide a safer and more effective strategy to modulate NLRP3-driven inflammation in DKD.

This study has several limitations. First, larger multi-ethnic cohorts with comprehensive clinical records and longitudinal follow-up will be needed to better define dynamic changes in SIRT6. Second, we did not perform a systematic evaluation of other HDAC classes, which should be addressed in future studies to provide a broader epigenetic perspective. Third, although our mouse models captured key features of DKD, they cannot fully recapitulate human heterogeneity [64], further validation in additional models and human-derived organoid systems will be essential to bridge this gap.

In conclusion, this study demonstrates that the histone deacetylase SIRT6 represses NLRP3 expression through H3K9 deacetylation, thereby protecting TECs and mitigating DKD progression. These findings establish SIRT6 as a direct epigenetic suppressor of NLRP3 and highlight its potential as a novel, translationally relevant therapeutic target in DKD. Taken together, our findings highlight epigenetic repression of inflammatory signaling as a central mechanism in DKD progression and point to SIRT6 as a potential entry point for stage-specific risk prediction and individualized intervention.

## Funding information

This study was supported by the Beijing Research Ward Construction Clinical Research Project (2022-YJXBF-04-02), National Natural Science Foundation of China (82170817, 81970713, 82205094, 82374419), Elite Medical Professionals Project of China-Japan Friendship Hospital (NO. ZRJY2024-BJ03) and National High-Level Hospital Clinical Research Funding (2023-NHLHCRF-DJZD-01, 2024-NHLHCRFJBGS-WZ-08 and 2025-NHLHCRF-JBGS-A-WZ-17).

## Compliance with ethics requirements

**Human renal tissue**

Renal biopsy samples from patients with DKD and histologically normal renal tissue (from tumor nephrectomy margins) were collected from the Department of Nephrology, China-Japan Friendship Hospital under approved protocols (2024-KY-129-1) All control specimens were rigorously screened to exclude pre-existing renal pathology. Comprehensive clinical and biochemical characteristics of both cohorts are presented in Tab. S1, 2. All DKD biopsies were histologically confirmed by certified renal pathologists and classified according to Kidney Disease: Improving Global Outcomes (KDIGO) eGFR-based guidelines. This study adhered to the ethical principles of the Declaration of Helsinki.

**Animals**

All animal experiments were performed in compliance with ARRIVE guidelines 2.0 and approved by the Institutional Animal Care and Use Committee (IACUC) of China-Japan Friendship Hospital (Protocol #ZRDWLL230011). Male littermate mice (C57BL/6J background) were randomly assigned to experimental groups using a blinded design. Animals were maintained under specific pathogen-free (SPF) conditions at a controlled temperature (22–24 °C) with a 12-hour light/dark cycle. Autoclaved cages, bedding, and irradiated food and water are provided ad libitum. Male mice were selected based on their well-documented metabolic susceptibility in prior studies, minimizing potential sex-specific variability.

## Declaration of competing interest

The authors declare that they have no known competing financial interests or personal relationships that could have appeared to influence the work reported in this paper.

## References

[1] C.K.D.W.G. Kidney Disease: Improving Global Outcomes, KDIGO 2024 Clinical practice guideline for the evaluation and management of chronic kidney disease. Kidney Int 2024;105:S117–S314. 10.1016/j.kint.2023.10.018. 10.1016/j.kint.2023.10.018 38490803

[2] Herrington W.G., Haynes R. (2024). Diabetic kidney disease - semaglutide flows into the mainstream. N Engl J Med.

[3] Li L., Liu Y. (2025). Podocyte aging and diabetic kidney disease. Kidney Int.

[4] Mori Y, Ajay AK, Chang JH, Mou S, Zhao H, Kishi S, Li J, Brooks CR, Xiao S, Woo HM, Sabbisetti VS, Palmer SC, Galichon P, Li L, Henderson JM, Kuchroo VK, Hawkins J, Ichimura T, Bonventre JV. KIM-1 mediates fatty acid uptake by renal tubular cells to promote progressive diabetic kidney disease. Cell Metab. 2021;33:1042–1061 e7. 10.1016/j.cmet.2021.04.004. 10.1016/j.cmet.2021.04.004 PMC8132466 33951465

[5] Tuttle K.R. (2023). Digging deep into cells to find mechanisms of kidney protection by SGLT2 inhibitors. J Clin Invest.

[6] Wu J., Sun Z., Yang S., Fu J., Fan Y., Wang N. (2022). Kidney single-cell transcriptome profile reveals distinct response of proximal tubule cells to SGLT2i and ARB treatment in diabetic mice. Mol Ther.

[7] Brancolini C., Gagliano T., Minisini M. (2022). HDACs and the epigenetic plasticity of cancer cells: Target the complexity. Pharmacol Ther.

[8] Perico L., Remuzzi G., Benigni A. (2024). Sirtuins in kidney health and disease. Nat Rev Nephrol.

[9] Wu X., Liu H., Brooks A., Xu S., Luo J., Steiner R. (2022). SIRT6 mitigates heart failure with preserved ejection fraction in diabetes. Circ Res.

[10] Li B., Xin Z., Gao S., Li Y., Guo S., Fu Y. (2023). SIRT6-regulated macrophage efferocytosis epigenetically controls inflammation resolution of diabetic periodontitis. Theranostics.

[11] Kang P., Xiao L., Liu Y., Yang J., Li S., Wang L. (2025). Morusin ameliorates tubulointerstitial damage in diabetic mice through SIRT1/HIF-1alpha/IL-16 signaling pathway. Phytomedicine.

[12] Deng Z., He M., Hu H., Zhang W., Zhang Y., Ge Y. (2024). Melatonin attenuates sepsis-induced acute kidney injury by promoting mitophagy through SIRT3-mediated TFAM deacetylation. Autophagy.

[13] Yuan Y., Yuan L., Yang J., Liu F., Liu S., Li L. (2024). Autophagy-deficient macrophages exacerbate cisplatin-induced mitochondrial dysfunction and kidney injury via miR-195a-5p-SIRT3 axis. Nat Commun.

[14] Liu M., Liang K., Zhen J., Zhou M., Wang X., Wang Z. (2017). Sirt6 deficiency exacerbates podocyte injury and proteinuria through targeting Notch signaling. Nat Commun.

[15] Kropp M., Golubnitschaja O., Mazurakova A., Koklesova L., Sargheini N., Vo T.K.S. (2023). Diabetic retinopathy as the leading cause of blindness and early predictor of cascading complications-risks and mitigation. EPMA J.

[16] Kropp M., De Clerck E., Vo T.K.S., Thumann G., Costigliola V., Golubnitschaja O. (2023). Short communication: unique metabolic signature of proliferative retinopathy in the tear fluid of diabetic patients with comorbidities - preliminary data for PPPM validation. EPMA J.

[17] Smokovski I., Steinle N., Behnke A., Bhaskar S.M.M., Grech G., Richter K. (2024). position. EPMA J.

[18] Kaya-Okur H.S., Wu S.J., Codomo C.A., Pledger E.S., Bryson T.D., Henikoff J.G. (2019). CUT&Tag for efficient epigenomic profiling of small samples and single cells. Nat Commun.

[19] Zhang Y., Qiao Y., Li Z., Liu D., Jin Q., Guo J. (2024). Intestinal NSD2 aggravates nonalcoholic steatohepatitis through histone modifications. Adv Sci (Weinh).

[20] Mise K., Long J., Galvan D.L., Ye Z., Fan G., Sharma R. (1965). NDUFS4 regulates cristae remodeling in diabetic kidney disease. Nat Commun.

[21] Kishi S., Brooks C.R., Taguchi K., Ichimura T., Mori Y., Akinfolarin A. (2019). Proximal tubule ATR regulates DNA repair to prevent maladaptive renal injury responses. J Clin Invest.

[22] Tuder R.M., Gandjeva A., Williams S., Kumar S., Kheyfets V.O., Hatton-Jones K.M. (2024). Digital spatial profiling identifies distinct molecular signatures of vascular lesions in pulmonary arterial hypertension. Am J Respir Crit Care Med.

[23] Y. Fu, Y. Sun, M. Wang, Y. Hou, W. Huang, D. Zhou, Z. Wang, S. Yang, W. Tang, J. Zhen, Y. Li, X. Wang, M. Liu, Y. Zhang, B. Wang, G. Liu, X. Yu, J. Sun, C. Zhang, F. Yi, Elevation of JAML Promotes Diabetic Kidney Disease by Modulating Podocyte Lipid Metabolism, Cell Metab. 32(2020) 1052-1062 e8. 10.1016/j.cmet.2020.10.019. 10.1016/j.cmet.2020.10.019 33186558

[24] Li Y., Duan Y., Chu Q., Lv H., Li J., Guo X. (2025). G-protein coupled receptor GPR124 protects against podocyte senescence and injury in diabetic kidney disease. Kidney Int.

[25] Lanaspa M.A., Ishimoto T., Cicerchi C., Tamura Y., Roncal-Jimenez C.A., Chen W. (2014). Endogenous fructose production and fructokinase activation mediate renal injury in diabetic nephropathy. J Am Soc Nephrol.

[26] Ginley B., Lutnick B., Jen K.Y., Fogo A.B., Jain S., Rosenberg A. (2019). Computational segmentation and classification of diabetic glomerulosclerosis. J Am Soc Nephrol.

[27] Xie Y, JE, Cai H, Zhong F, Xiao W, Gordon RE, Wang L, Zheng YL. Zhang A, Lee K, He JC. Reticulon-1A mediates diabetic kidney disease progression through endoplasmic reticulum-mitochondrial contacts in tubular epithelial cells. Kidney Int 2022;102:293–306. 10.1016/j.kint.2022.02.038. 10.1016/j.kint.2022.02.038 PMC9329239 35469894

[28] Huang R., Fu P., Ma L. (2023). Kidney fibrosis: from mechanisms to therapeutic medicines. Signal Transduct Target Ther.

[29] Shahzad K., Fatima S., Khawaja H., Elwakiel A., Gadi I., Ambreen S. (2022). Podocyte-specific Nlrp3 inflammasome activation promotes diabetic kidney disease. Kidney Int.

[30] Zhang C., Huang Y., Ouyang F., Su M., Li W., Chen J. (2022). Extracellular vesicles derived from mesenchymal stem cells alleviate neuroinflammation and mechanical allodynia in interstitial cystitis rats by inhibiting NLRP3 inflammasome activation. J Neuroinflammation.

[31] Wu M., Yang Z., Zhang C., Shi Y., Han W., Song S. (2021). Inhibition of NLRP3 inflammasome ameliorates podocyte damage by suppressing lipid accumulation in diabetic nephropathy. Metabolism.

[32] Tang G., Li S., Zhang C., Chen H., Wang N., Feng Y. (2021). Clinical efficacies, underlying mechanisms and molecular targets of Chinese medicines for diabetic nephropathy treatment and management. Acta Pharm Sin B.

[33] Barrera-Chimal J., Lima-Posada I., Bakris G.L., Jaisser F. (2022). Mineralocorticoid receptor antagonists in diabetic kidney disease - mechanistic and therapeutic effects. Nat Rev Nephrol.

[34] Tang M., Morieri M.L., Kalim S., Doria A. (2025). Combination therapy with SGLT2 inhibitors and GLP-1 receptor agonists for diabetic kidney disease. J Am Soc Nephrol.

[35] Fioretto P., Zambon A., Rossato M., Busetto L., Vettor R. (2016). SGLT2 inhibitors and the diabetic kidney. Diabetes Care.

[36] Li C., Chen K., Fang Q., Shi S., Nan J., He J. (2024). Crosstalk between epitranscriptomic and epigenomic modifications and its implication in human diseases. Cell Genom.

[37] Mimura I., Chen Z., Natarajan R. (2025). Epigenetic alterations and memory: key players in the development/progression of chronic kidney disease promoted by acute kidney injury and diabetes. Kidney Int.

[38] Xiao Y., Xiao X., Zhang X., Yi D., Li T., Hao Q. (2024). Mediterranean diet in the targeted prevention and personalized treatment of chronic diseases: evidence, potential mechanisms, and prospects. EPMA J.

[39] Golubnitschaja O., Polivka J., Potuznik P., Pesta M., Stetkarova I., Mazurakova A. (2024). The paradigm change from reactive medical services to 3PM in ischemic stroke: a holistic approach utilising tear fluid multi-omics, mitochondria as a vital biosensor and AI-based multi-professional data interpretation. EPMA J.

[40] Golubnitschaja O., Kapinova A., Sargheini N., Bojkova B., Kapalla M., Heinrich L. (2024). Mini-encyclopedia of mitochondria-relevant nutraceuticals protecting health in primary and secondary care-clinically relevant 3PM innovation. EPMA J.

[41] Zhang Y., Yang Y., Yang F., Liu X., Zhan P., Wu J. (2023). HDAC9-mediated epithelial cell cycle arrest in G2/M contributes to kidney fibrosis in male mice. Nat Commun.

[42] Katsyuba E., Romani M., Hofer D., Auwerx J. (2020). NAD(+) homeostasis in health and disease. Nat Metab.

[43] Sun H.J., Xiong S.P., Cao X., Cao L., Zhu M.Y., Wu Z.Y. (2021). Polysulfide-mediated sulfhydration of SIRT1 prevents diabetic nephropathy by suppressing phosphorylation and acetylation of p65 NF-kappaB and STAT3. Redox Biol.

[44] Li S., Lin Z., Xiao H., Xu Z., Li C., Zeng J. (2023). Fyn deficiency inhibits oxidative stress by decreasing c-Cbl-mediated ubiquitination of Sirt1 to attenuate diabetic renal fibrosis. Metabolism.

[45] Su P.P., Liu D.W., Zhou S.J., Chen H., Wu X.M., Liu Z.S. (2022). Down-regulation of Risa improves podocyte injury by enhancing autophagy in diabetic nephropathy. Mil Med Res.

[46] Yasuda I., Hasegawa K., Sakamaki Y., Muraoka H., Kawaguchi T., Kusahana E. (2021). Pre-emptive short-term nicotinamide mononucleotide treatment in a mouse model of diabetic nephropathy. J Am Soc Nephrol.

[47] Lv Z., Hu J., Su H., Yu Q., Lang Y., Yang M. (2025). TRAIL induces podocyte PANoptosis via death receptor 5 in diabetic kidney disease. Kidney Int.

[48] Smith K.D., Prince D.K., Henriksen K.J., Nicosia R.F., Alpers C.E., Akilesh S. (2022). Digital spatial profiling of collapsing glomerulopathy. Kidney Int.

[49] Roichman A., Elhanati S., Aon M.A., Abramovich I., Di Francesco A., Shahar Y. (2021). Restoration of energy homeostasis by SIRT6 extends healthy lifespan. Nat Commun.

[50] Guo J, Wang Z, Wu J, Liu M, Li M, Sun Y, Huang W, Li Y, Zhang Y, Tang W, Li X, Zhang C, Hong F, Li N, Nie J, Yi F. Endothelial SIRT6 is vital to prevent hypertension and associated cardiorenal injury through targeting Nkx3.2-GATA5 signaling. Circ Res 2019;124:1448-1461.10.1161/CIRCRESAHA.118.314032. 10.1161/CIRCRESAHA.118.314032 30894089

[51] Li Z., Xu K., Zhang N., Amador G., Wang Y., Zhao S. (2018). Overexpressed SIRT6 attenuates cisplatin-induced acute kidney injury by inhibiting ERK1/2 signaling. Kidney Int.

[52] Cai J., Liu Z., Huang X., Shu S., Hu X., Zheng M. (2020). The deacetylase sirtuin 6 protects against kidney fibrosis by epigenetically blocking beta-catenin target gene expression. Kidney Int.

[53] Zheng Y., Zhang T.N., Hao P.H., Yang N., Du Y. (2025). Histone deacetylases and their inhibitors in kidney diseases. Mol Ther.

[54] Jiang W.J., Xu C.T., Du C.L., Dong J.H., Xu S.B., Hu B.F. (2022). Tubular epithelial cell-to-macrophage communication forms a negative feedback loop via extracellular vesicle transfer to promote renal inflammation and apoptosis in diabetic nephropathy. Theranostics.

[55] Janssens D.H., Meers M.P., Wu S.J., Babaeva E., Meshinchi S., Sarthy J.F. (2021). Automated CUT&Tag profiling of chromatin heterogeneity in mixed-lineage leukemia. Nat Genet.

[56] Coll R.C., Schroder K. (2025). Inflammasome components as new therapeutic targets in inflammatory disease. Nat Rev Immunol.

[57] Wang C, Chen Q, Chen S, Fan L, Gan Z, Zhao M, Shi L, Bin P, Yang G, Zhou X, Ren W. Serine synthesis sustains macrophage IL-1beta production via NAD(+)-dependent protein acetylation. Mol Cell 2024;84:744-759 e6. 10.1016/j.molcel.2024.01.002. 10.1016/j.molcel.2024.01.002 38266638

[58] He M, Chiang HH, Luo H, Zheng Z, Qiao Q, Wang L, Tan M, Ohkubo R, Mu WC, Zhao S, Wu H, Chen D. An acetylation switch of the NLRP3 inflammasome regulates aging-associated chronic inflammation and insulin resistance. Cell Metab 2020;31:580–591 e5. 10.1016/j.cmet.2020.01.009. 10.1016/j.cmet.2020.01.009 PMC7104778 32032542

[59] Zhang Y., Luo L., Xu X., Wu J., Wang F., Lu Y. (2023). Acetylation is required for full activation of the NLRP3 inflammasome. Nat Commun.

[60] Huang Z., Zhao J., Deng W., Chen Y., Shang J., Song K. (2018). Identification of a cellularly active SIRT6 allosteric activator. Nat Chem Biol.

[61] Collins J.A., Kim C.J., Coleman A., Little A., Perez M.M., Clarke E.J. (2023). Cartilage-specific Sirt6 deficiency represses IGF-1 and enhances osteoarthritis severity in mice. Ann Rheum Dis.

[62] Feng S., Wierzbowski M.C., Hrovat-Schaale K., Dumortier A., Zhang Y., Zyulina M. (2025). Mechanisms of NLRP3 activation and inhibition elucidated by functional analysis of disease-associated variants. Nat Immunol.

[63] Coll R.C., Hill J.R., Day C.J., Zamoshnikova A., Boucher D., Massey N.L. (2019). MCC950 directly targets the NLRP3 ATP-hydrolysis motif for inflammasome inhibition. Nat Chem Biol.

[64] Azushima K., Gurley S.B., Coffman T.M. (2018). Modelling diabetic nephropathy in mice. Nat Rev Nephrol.

