# Supplementary material for: Mitigation of renal tubular injury by SIRT6 may improve individual outcomes in diabetic kidney disease-potential mechanisms involving epigenetic repression of inflammatory responses
Source: J Adv Res. 2025 Oct 10;85:281–94. doi: 10.1016/j.jare.2025.10.008 (PMC13316547; doi:10.1016/j.jare.2025.10.008)
Supplement: Supplementary Data 1 [file mmc1.docx]

**Mitigation of renal tubular injury by SIRT6 may improve individual outcomes in diabetic kidney disease– potential mechanisms involving epigenetic repression of inflammatory responses**

**Supplementary Materials and Methods.**

**STZ/HFD-induced DKD model**

To establish a robust DKD model, 4-week-old male *Sirt6*^fl/fl^ and *Sirt6*^ΔTEC^ mice (C57BL/6J background) were fed either a HFD (60% kcal fat) (#D12492, Research Diets) or CD (#D12450J, Research Diets) for 4 weeks. After uninephrectomy, mice were allowed a 1-week recovery period, followed by induction of insulin deficiency via low-dose STZ (50 mg/kg/day for 4 days, pH 4.5 citrate buffer) (#572201, Sigmal). Mice were then maintained on HFD for an additional 14 weeks (total 24 weeks)[1]. At 10 weeks, mice were randomized into four treatment groups: vehicle control or MCC950 (10 mg/kg, i. p. every 3 days)[2, 3]. Terminal analyses at 24 weeks included biochemical assays of blood/urine, histopathology, and molecular profiling of renal cortical tissues.

***db/db* mice**

Male *db/db* mice and their non-diabetic *db/m* littermate controls were purchased from Cyagen (C001274). The *db/db* mice exhibited progressive metabolic disturbances, including obesity onset at 3-4 weeks of age, followed by hyperglycemia (4-8 weeks) and proteinuria (8-20 weeks), confirming successful establishment of type 2 diabetic kidney disease. Age-matched *db/m* mice served as genetic controls[1].

**AAV9-mediated *Sirt6* overexpression in renal TECs**

We generated AAV9 vectors carrying mouse *Sirt6* (NM_001163430.2) under a TEC-specific promoter (*Ksp*) with luciferase-T2A reporter[4]. After packaging in HEK293 cells and purification, *db/m* and *db/db* mice received 1×10^^11^ viral genomes via tail vein injection. TEC-specific *Sirt6* overexpression was confirmed 4-6 weeks post-injection by western blot, and IHC[5].

**SIRT6 activation studies**

Starting at 24 weeks of age, *db/m* and *db/db* mice were randomly allocated to receive either MDL-800 (10 mg/kg, intraperitoneally every 3 days) or vehicle control for 8 weeks until the experimental endpoint. The dosing regimen was based on previous studies in which MDL-800 was shown to effectively activate SIRT6 without overt toxicity at the applied dose[6].

**Cell culture and treatments**

HK-2 cells (#BFN60700259, ATCC) (Research Resource Identifiers (RRIDs): CVCL_0302) were maintained in DMEM/F12 (#21331020, Gibco) supplemented with 10% FBS (#A5670701, Gibco) and 1% penicillin/streptomycin (#15140122, Gibco). Following 6h serum starvation for synchronization, subsequently, cells were exposed to either: (1) HG (30 mM D-glucose, #G8270, Sigma) versus normal glucose control (5.6 mM), or (2) MCC950 (10 uM, #HY-12815, MCE)[3].

**Histological analysis of renal tissues**

Renal tissues from human biopsies and mouse models were fixed overnight in 4% paraformaldehyde, embedded in paraffin, and sectioned (4um). IHC was performed as previously described. Sections were stained with H&E, Masson's trichrome, and PAS using commercial kits (#G1120, #G1340, #G1281, Solarbio) following manufacturer's protocols. For quantification, at least 6 random fields per human sample or 10 fields per mouse section were imaged at 50x magnification (Nikon, Japan)[7].

**RNA extraction and quantitative real‑time PCR analysis**

Total RNA was isolated from tissues or cells using TRIzol reagent (#15596018CN, Invitrogen). cDNA was synthesized from 1 ug of total RNA using HiScript III RT SuperMix (#R333, Vazyme). Quantitative PCR was performed with Taq Pro Universal SYBR Master Mix (#Q712, Vazyme) on a Bio-Rad iCycler, with β-actin serving as the internal control. Relative gene expression was calculated using the 2-^ΔΔCT^ method and expressed as fold change compared to controls. Primer sequences are listed in Tab. S3.

**Western blot analyses**

Tissues and cells were lysed in RIPA buffer (#P0013B, Beyotime) supplemented with protease (#P1010, Beyotime) and deacetylase (#P1112, Beyotime) inhibitors, homogenized, and centrifuged (12,000 rpm, 15 min, 4℃). Protein concentrations were quantified using a BCA assay (#P0012S, Beyotime). Equal amounts of protein were resolved by 10% SDS-PAGE, transferred to PVDF membranes (#PVH00010, Millipore), and probed with target-specific antibodies. GAPDH served as the loading control. Antibody details are listed in Tab. S4, and full blots are shown in Fig. S6-10.

**Immunoflurescence Staining**

Tissue samples were fixed in 4% PFA at 4℃ overnight, embedded in paraffin, and sectioned at 4 um thickness. Immunofluorescence staining was performed following an established protocol[8], using primary antibodies detailed in Table S4. Images were acquired using microscope system (ZEISS, Germany).

**TUNEL Assay**

Cell death in different intervention settings was detected by TUNEL assay following the manufacturer’s protocol (#12156792910, Roche Diagnostics).

**Urinary Biomarker Analysis**

Urinary albumin was quantified by ELISA (ELISA, #E-EL-M0792, Elabscience), while creatinine (colorimetric assay, #E-BC-K188-M, Elabscience) and NAG (ELISA, #CS0780, Sigma-Aldrich) levels were measured in parallel. Data were normalized as UACR and urinary NAG/Cr[9, 10].

**TEM**

Kidney tissues were fixed in 2.5% glutaraldehyde, post-fixed with 1% osmium tetroxide, and dehydrated through a graded ethanol series. Samples were then embedded in epoxy resin, and ultrathin sections (70 nm) were prepared using an ultramicrotome. Imaging was performed on a Hitachi HT-7800 microscope (operated at 80 kV) at the Core Electron Microscopy Laboratory, China-Japan Friendship Hospital. Glomerular GBM thickness and podocyte foot process width are quantified from 50 randomly acquired electron micrographs (5 glomeruli per mouse, 10 images per glomerulus) using ImageJ software with curvilinear analysis.

**SEM**

Scanning electron microscopy (SEM) was performed as previously described with minor modifications. Briefly, tissue samples were fixed in 3% glutaraldehyde and 2% paraformaldehyde in 0.1 M cacodylate buffer (pH 7.3), followed by washing in 0.1 M cacodylate buffer. Samples were then postfixed in 1% osmium tetroxide (OsO_4_) in cacodylate buffer, rinsed with distilled water, and sequentially treated with 1% aqueous tannic acid (Millipore-filtered) and 1% aqueous uranyl acetate (Millipore-filtered), with thorough distilled water washes between steps.Dehydration was carried out using a graded ethanol series, followed by incubation in increasing concentrations of hexamethyldisilazane (HMDS) and air-drying overnight. Dried samples were mounted on double-stick carbon tabs (Ted Pella) affixed to glass slides, then sputter-coated with a 25-nm platinum alloy layer using a Balzer MED 010 evaporator (Technotrade International) and immediately flash-coated with carbon under vacuum. Samples were stored in a desiccator prior to imaging. SEM was performed using a JSM-5910 microscope (JEOL) at an accelerating voltage of 5 kV.

**siRNA-Mediated Knockdown**

Cells were maintained in antibiotic-free culture medium (#11058021 Gibco) until they reached the desired confluence. Transfections were performed using Lipofectamine^TM^ 3000 reagent (#L3000001, Thermo Fisher Scientific) according to the supplier’s protocol. Target-specific siRNAs or the corresponding scramble negative controls, were introduced into the cells under these conditions.

**RNA-seq Analysis**

Total RNA was isolated from TECs using TRIzol reagent (#15596018CN, Invitrogen), with RNA quality verified by NanoDrop 2000 (Thermo Fisher Scientific) and Agilent 2100 Bioanalyzer systems. Sequencing libraries were prepared with the VAHTS Universal V6 RNA-seq Kit (Vazyme) and paired-end sequencing was performed on an Illumina NovaSeq 6000 platform (OE Biotech, Shanghai)[8].

**CHIP**

ChIP assays were performed using the BeyoChIPTM Enzymatic Kit (#P2083S, Beyotime) with isotype IgG controls. Immunoprecipitations employed anti-H3K9ac (#A22565, 1:2000, ABclonal), anti-RNAP2 (#MA1-46093, 1:100, Invitrogen), and control IgG (#30000-0-AP, 1:300, Proteintech). Precipitated DNA was analyzed by PCR or qPCR with gene-specific primers (Tab. S3)[8].

**Dual-luciferase reporter assay**

HK-2 cells were plated in 24-well plates (5×10^4^ cells/well) and co-transfected with a firefly luciferase reporter plasmid harboring the SIRT6 promoter (200 ng), either KLF15-overexpressing or control plasmid (200 ng), and the Renilla luciferase internal control pRL-TK (50 ng). Primer sequences are listed in Tab. S3. After 24 hours, cells were lysed, and luminescence was quantified using the Dual-Glo Luciferase Assay System (#E5311, Promega) on a GloMax 20/20 Luminometer. Firefly luciferase activity was normalized to Renilla for data analysis.

**References**

[1] Y. Fu, Y. Sun, M. Wang, Y. Hou, W. Huang, D. Zhou, Z. Wang, S. Yang, W. Tang, J. Zhen, Y. Li, X. Wang, M. Liu, Y. Zhang, B. Wang, G. Liu, X. Yu, J. Sun, C. Zhang, F. Yi, Elevation of JAML Promotes Diabetic Kidney Disease by Modulating Podocyte Lipid Metabolism, Cell Metab. 32(2020) 1052-1062 e8.10.1016/j.cmet.2020.10.019.

[2] M. Wu, Z. Yang, C. Zhang, Y. Shi, W. Han, S. Song, L. Mu, C. Du, Y. Shi, Inhibition of NLRP3 inflammasome ameliorates podocyte damage by suppressing lipid accumulation in diabetic nephropathy, Metabolism. 118(2021) 154748.10.1016/j.metabol.2021.154748.

[3] C. Zhang, Y. Huang, F. Ouyang, M. Su, W. Li, J. Chen, H. Xiao, X. Zhou, B. Liu, Extracellular vesicles derived from mesenchymal stem cells alleviate neuroinflammation and mechanical allodynia in interstitial cystitis rats by inhibiting NLRP3 inflammasome activation, J Neuroinflammation. 19(2022) 80.10.1186/s12974-022-02445-7.

[4] S. Schievenbusch, I. Strack, M. Scheffler, R. Nischt, O. Coutelle, M. Hosel, M. Hallek, J.W. Fries, H.P. Dienes, M. Odenthal, H. Buning, Combined paracrine and endocrine AAV9 mediated expression of hepatocyte growth factor for the treatment of renal fibrosis, Mol Ther. 18(2010) 1302-9.10.1038/mt.2010.71.

[5] Y. Zhao, X. Jia, X. Yang, X. Bai, Y. Lu, L. Zhu, W. Cheng, M. Shu, Y. Zhu, X. Du, L. Wang, Y. Shu, Y. Song, S. Jin, Deacetylation of Caveolin-1 by Sirt6 induces autophagy and retards high glucose-stimulated LDL transcytosis and atherosclerosis formation, Metabolism. 131(2022) 155162.10.1016/j.metabol.2022.155162.

[6] X. Wu, H. Liu, A. Brooks, S. Xu, J. Luo, R. Steiner, D.M. Mickelsen, C.S. Moravec, A.D. Jeffrey, E.M. Small, Z.G. Jin, SIRT6 Mitigates Heart Failure With Preserved Ejection Fraction in Diabetes, Circ Res. 131(2022) 926-943.10.1161/CIRCRESAHA.121.318988.

[7] X. Liu, Y. Zhang, Y. Wang, Y. Yang, Z. Qiao, P. Zhan, H. Jin, Q. Xu, W. Tang, Y. Sun, Y. Zhang, F. Yi, M. Liu, Tubular MYDGF Slows Progression of Chronic Kidney Disease by Maintaining Mitochondrial Homeostasis, Adv Sci (Weinh). 12(2025) e2409756.10.1002/advs.202409756.

[8] Y. Zhang, Y. Qiao, Z. Li, D. Liu, Q. Jin, J. Guo, X. Li, L. Chen, L. Liu, L. Peng, Intestinal NSD2 Aggravates Nonalcoholic Steatohepatitis Through Histone Modifications, Adv Sci (Weinh). 11(2024) e2402551.10.1002/advs.202402551.

[9] Y. Li, Y. Duan, Q. Chu, H. Lv, J. Li, X. Guo, Y. Gao, M. Liu, W. Tang, H. Hu, H. Liu, J. Sun, X. Wang, F. Yi, G-protein coupled receptor GPR124 protects against podocyte senescence and injury in diabetic kidney disease, Kidney Int. 107(2025) 652-665.10.1016/j.kint.2024.12.013.

[10] M.A. Lanaspa, T. Ishimoto, C. Cicerchi, Y. Tamura, C.A. Roncal-Jimenez, W. Chen, K. Tanabe, A. Andres-Hernando, D.J. Orlicky, E. Finol, S. Inaba, N. Li, C.J. Rivard, T. Kosugi, L.G. Sanchez-Lozada, J.M. Petrash, Y.Y. Sautin, A.A. Ejaz, W. Kitagawa, G.E. Garcia, D.T. Bonthron, A. Asipu, C.P. Diggle, B. Rodriguez-Iturbe, T. Nakagawa, R.J. Johnson, Endogenous fructose production and fructokinase activation mediate renal injury in diabetic nephropathy, J Am Soc Nephrol. 25(2014) 2526-38.10.1681/ASN.2013080901.

**Tab. S1 General patients’ data of DSP**

| Sample ID | Groups | Gender | Age | Years of diabetes | BP (mmHg) | SCr  (umol/L) | BUN (mmol/L) | eGFR (ml/min/1.73m2） | 24h urinary protein excretion (g) | BMI（kg/m2） | FBG (mmol/L) | HbA1c(%) |
| --- | --- | --- | --- | --- | --- | --- | --- | --- | --- | --- | --- | --- |
| 1 | Normal | female | 36 | 0 | 125/85 | 76 | 3.24 | 90 | - | 20.57 | 4.10 | 3.10 |
| 2 | Normal | female | 40 | 0 | 134/90 | 73 | 3.55 | 92.1 | - | 19.82 | 3.20 | 3.80 |
| 3 | Normal | male | 63 | 0 | 148/87 | 75.5 | 4.22 | 97.7 | - | 26.99 | 4.50 | 2.70 |
| 4 | Normal | male | 38 | 0 | 143/72 | 73 | 4.85 | 115.3 | - | 20.31 | 3.80 | 3.20 |
| 5 | Normal | female | 72 | 0 | 117/63 | 56.1 | 5.35 | 94.1 | - | 25.91 | 5.44 | 2.80 |
| 6 | Normal | female | 51 | 0 | 126/76 | 69.3 | 13.89 | 91.6 | - | 21.22 | 5.74 | 3.40 |
| 7 | Normal | female | 30 | 0 | 131/82 | 61 | 3.07 | 119.7 | - | 19.49 | 5.43 | 3.30 |
| 8 | Normal | male | 40 | 0 | 126/86 | 62.2 | 4.31 | 119.5 | - | 21.22 | 5.74 | 2.40 |
| 9 | Normal | male | 65 | 0 | 122/71 | 69.5 | 10.11 | 98.9 | - | 21.10 | 3.04 | 2.70 |
| 10 | Normal | female | 67 | 0 | 132/80 | 40.6 | 4.59 | 104.9 | - | 19.71 | 2.94 | 2.50 |
| 11 | Normal | female | 61 | 0 | 140/71 | 41.5 | 3.51 | 108.3 | - | 19.63 | 4.09 | 2.80 |
| 12 | Normal | male | 40 | 0 | 117/80 | 70 | 6.7 | 115.3 | - | 23.78 | 4.99 | 3.50 |
| 13 | Normal | male | 53 | 0 | 125/79 | 78.9 | 4.93 | 102.6 | - | 29.41 | 5.42 | 3.60 |
| 14 | DKD | male | 63 | 23 | 188/104 | 142.9 | 13.46 | 47.8 | 0.62 | 24.22 | 3.02 | 4.90 |
| 15 | DKD | female | 63 | 13 | 135/86 | 163.2 | 15 | 30.4 | 2.73 | 28.20 | 5.82 | 6.00 |
| 16 | DKD | male | 43 | 20 | 173/102 | 163 | 8.12 | 46.3 | 4.53 | 24.77 | 4.69 | 7.30 |
| 17 | DKD | male | 41 | 1 | 143/82 | 159.3 | 14.98 | 48.1 | 2.47 | 36.33 | 5.38 | 6.50 |
| 18 | DKD | female | 60 | 15 | 126/71 | 82.1 | 4.93 | 70.6 | 4.04 | 24.52 | 6.63 | 8.70 |
| 19 | DKD | male | 43 | 0.5 | 117/78 | 163.4 | 10.19 | 46.1 | 2.72 | 25.16 | 7.29 | 6.80 |
| 20 | DKD | male | 74 | 20 | 126/60 | 213.3 | 12.78 | 27.6 | 3.78 | 25.47 | 12.07 | 8.50 |
| 21 | DKD | male | 54 | 9 | 126/77 | 90.9 | 6.42 | 87.1 | 2.1 | 25.35 | 4.58 | 6.40 |
| 22 | DKD | male | 52 | 5 | 129/75 | 204.7 | 11.03 | 33.3 | 2.42 | 26.85 | 3.89 | 4.90 |
| 23 | DKD | male | 45 | 12 | 130/86 | 192.5 | 10.03 | 37.4 | 1.16 | 22.49 | 4.05 | 4.90 |
| 24 | DKD | female | 66 | 24 | 129/73 | 206.3 | 16.8 | 22.5 | 1.72 | 25.07 | 4.78 | 9.90 |
| 25 | DKD | male | 32 | 8 | 130/86 | 113.6 | 8.27 | 76.4 | 3.13 | 24.78 | 5.04 | 5.90 |
| 26 | DKD | male | 64 | 20 | 136/75 | 111 | 7.33 | 72.9 | 1.52 | 23.74 | 8.68 | 8.50 |
| 27 | DKD | male | 44 | 15 | 153/87 | 100.7 | 8.09 | 81.9 | 2.59 | 26.06 | 7.60 | 7.70 |
| 28 | DKD | male | 57 | 10 | 126/69 | 90.2 | 8.52 | 87.3 | 1.8 | 23.67 | 4.96 | 7.10 |
| 29 | DKD | male | 55 | 5 | 120/80 | 98.4 | 6.19 | 48.7 | 1.95 | 21.36 | 6.16 | 6.90 |
| 30 | DKD | male | 55 | 15 | 125/81 | 134.8 | 14.16 | 51.6 | 1.009 | 20.62 | 4.47 | 6.20 |
| 31 | DKD | male | 62 | 4 | 149/82 | 87.4 | 7.81 | 64.7 | 1.99 | 22.86 | 5.00 | 5.80 |
| 32 | DKD | female | 62 | 16 | 121/75 | 99 | 14.97 | 55.7 | 0.96 | 27.34 | 5.38 | 6.70 |
| 33 | DKD | male | 38 | 10 | 120/80 | 81.7 | 5.37 | 73.8 | 4.12 | 30.86 | 7.41 | 6.20 |
| 34 | DKD | female | 54 | 10 | 140/60 | 183.9 | 7.42 | 27.9 | 1.5 | 29.30 | 9.64 | 6.50 |
| 35 | DKD | female | 62 | 10 | 165/75 | 129 | 13.95 | 54.1 | 2.9 | 28.58 | 12.10 | 5.20 |
| 36 | DKD | female | 49 | 23 | 138/84 | 87.8 | 6.12 | 69.8 | 0.85 | 25.63 | 7.15 | 7.60 |

**Tab. S2 General patients’ data of IHC**

| Sample ID | Groups | Gender | Age | SCr (umol/L) | eGFR (ml/min/1.73m2） | BUN (mmol/L) | 24h urinary protein excretion (g) |
| --- | --- | --- | --- | --- | --- | --- | --- |
| 1 | Normal | 33 | female | 97 | 91.8 | 7.04 | - |
| 2 | Normal | 42 | female | 65 | 104.6 | 5.35 | - |
| 3 | Normal | 34 | female | 64 | 112 | 4.53 | - |
| 4 | Normal | 41 | female | 72 | 93.1 | 6.77 | - |
| 5 | Normal | 35 | male | 97 | 90.6 | 6.67 | - |
| 6 | Normal | 34 | male | 70 | 119.7 | 3.53 | - |
| 7 | Normal | 40 | female | 70 | 96.9 | 5.22 | - |
| 8 | Normal | 38 | female | 50 | 119.5 | 4.78 | - |
| 9 | Normal | 40 | female | 74 | 90.6 | 6.89 | - |
| 10 | Normal | 43 | female | 57 | 112.2 | 4.98 | - |
| 11 | Normal | 40 | male | 67 | 116.8 | 5.44 | - |
| 12 | Normal | 54 | female | 62 | 102.7 | 4.57 | - |
| 13 | DKD | 44 | female | 83 | 77 | 7.17 | 3.58 |
| 14 | DKD | 69 | male | 97 | 73.4 | 7.12 | 0.84 |
| 15 | DKD | 41 | male | 132 | 60.3 | 7.56 | 1.05 |
| 16 | DKD | 69 | male | 120 | 56.8 | 8.07 | 1.3345 |
| 17 | DKD | 66 | male | 108 | 65.7 | 8.8 | 0.862 |
| 18 | DKD | 58 | female | 137 | 38.7 | 9.1 | 2.52 |
| 19 | DKD | 71 | female | 88 | 60.7 | 9.58 | 2.77 |
| 20 | DKD | 67 | male | 158 | 30.8 | 10.6 | 1.226 |
| 21 | DKD | 66 | male | 195 | 24.1 | 12.93 | 3.456 |
| 22 | DKD | 59 | male | 129 | 55.5 | 13.87 | 5.134 |
| 23 | DKD | 51 | male | 89 | 91 | 4.29 | 0.32 |
| 24 | DKD | 72 | male | 270 | 21.1 | 14.44 | 5.569 |
| 25 | DKD | 77 | male | 106 | 62.8 | 5.21 | 1.738 |
| 26 | DKD | 42 | male | 111 | 73.8 | 5.89 | 1.381 |
| 27 | DKD | 54 | female | 87 | 68.4 | 4.54 | 1.612 |
| 28 | DKD | 65 | male | 64 | 101.4 | 6.42 | 0.4 |
| 29 | DKD | 41 | female | 232 | 22.9 | 2.11 | 2.05 |
| 30 | DKD | 66 | female | 138 | 36.5 | 8.8 | 2.862 |
| 31 | DKD | 58 | male | 73 | 101.8 | 4.85 | 1.829 |
| 32 | DKD | 49 | female | 118 | 48.9 | 5.35 | 2.842 |
| 33 | DKD | 60 | male | 178 | 37.4 | 16.39 | 2.732 |
| 34 | DKD | 43 | female | 108 | 56.5 | 9.18 | 3.458 |
| 35 | DKD | 26 | male | 164 | 51 | 3.65 | 0.675 |
| 36 | DKD | 71 | female | 212 | 21.1 | 23.5 | 0.56 |
| 37 | DKD | 67 | female | 128 | 39.7 | 7.93 | 3.1 |
| 38 | DKD | 33 | male | 98 | 90.7 | 8 | 1.786 |

**Tab. S3 Main primers**

| Species | Gene | Sequence (5'-3') |
| --- | --- | --- |
| Mouse | β-actin-F | CCTCACTGTCCACCTTCC |
| Mouse | β-actin-R | GGGTGTAAAACGCAGCTC |
| Mouse | Sirt1-F | CAGCTCCTTTGCCACTATCC |
| Mouse | Sirt1-R | TGGCAACCTGACGATCAAG |
| Mouse | Sirt2-F | GCTGGAGGTGCTGAAGAAAC |
| Mouse | Sirt2-R | CAGGTCCAGGTCATCCAGAT |
| Mouse | Sirt3-F | TCCAGCCCTACACCATCTTC |
| Mouse | Sirt3-R | GCTCCAGGTAGCCATCTTCC |
| Mouse | Sirt4-F | GCTGCTGGAGAAGGAGAAGG |
| Mouse | Sirt4-R | CAGGTGGTGGTGGTGAAGAT |
| Mouse | Sirt5-F | CAGGTGCTGGAGAAGGAGAA |
| Mouse | Sirt5-R | GCTCCAGGTAGCCATCTTCC |
| Mouse | Sirt6-F | GCTGCTGGAGAAGGAGAAGG |
| Mouse | Sirt6-R | CAGGTGGTGGTGGTGAAGAT |
| Mouse | Sirt7-F | CAGCTCCTTTGCCACTATCC |
| Mouse | Sirt7-R | TGGCAACCTGACGATCAAG |
| Mouse | Sirt6-tail-F | GTCTTTGTTGTTTCTGAAGGGGTG |
| Mouse | Sirt6-tail-R | AAGATGCAGCTCTACTTGTCTAGG |
| Mouse | Sirt6-'2-3'-F | TCTTCGACCCACCAGAGGAG |
| Mouse | Sirt6-'2-3'-R | AAGCCCATGCGTTCTAGCTG |
| Mouse | Ggt1-Cre-F | GACGATGAAGCATGTTTAGCTGG |
| Mouse | Ggt1-Cre-R | CAGGGAAGATTGGCTGTGGGTT |
| Mouse | Cd44-F | GACACATATTGCTTCAATGCTTCAG |
| Mouse | Cd44-R | GATGCCAAGATGATCAGCCATT |
| Mouse | Tlr2-F | CTCCCAGGTAGGTCTTGGTAAA |
| Mouse | Tlr2-R | GAGGCGGACATCCTGAAC |
| Mouse | Nlrp3 (a site) -F | TGTCTTCTTCAAGGTCATTAGTGGCTT |
| Mouse | Nlrp3 (a site) -R | AGACAGGGAAAGCCTGTTGATAC |
| Mouse | Nlrp3 (b site) -F | ATGCCAGGAGAAAGAAATTAGAATCTAGG |
| Mouse | Nlrp3 (b site) -R | AGAAACAATGGGAAAAAGTGAAACCTC |
| Mouse | Nlrp3 (c site) -F | AGGAACTTTTCTTCCATGGCTCAGG |
| Mouse | Nlrp3 (c site) -R | TCTCCACTTCCAGAAAACGAAGTTATC |
| Mouse | Nlrp3 (d site) -F | TCTGCAGCTTAGGCTTACACTCAG |
| Mouse | Nlrp3 (d site) -R | TATATAGGGGTCACTGTGACACTGG |
| Mouse | Nlrp3 (e site) -F | TCTGTCCCTGTGAACTTTACCTCTAT |
| Mouse | Nlrp3 (e site) -R | GCACAGATGAGACAAGCACAATGTA |
| Human | β-actin-F | CATGTACGTTGCTATCCAGGC |
| Human | β-actin-R | CTCCTTAATGTCACGCACGAT |
| Human | Sirt6-F | GCCTCAGCAGGAGAAGATGA |
| Human | Sirt6-R | TCCAGGTGCTGAAGAGGAAG |
| Human | Nlrp3 (a site)-F | CAACCATAGTTTACACCCACGACTTC |
| Human | Nlrp3 (a site)-R | CTGCATCAGGATTCTGCCTTTCTTC |
| Human | Nlrp3 (b site)-F | GTGAGTCTGAGGCATCTTGACATTG |
| Human | Nlrp3 (b site)-R | AGAGTCCGGATGGATGAACAAGTTG |
| Human | Nlrp3 (c site)-F | CTGGGGAAGTGTGTCTTTTAGTCATCTA |
| Human | Nlrp3 (c site)-R | AGGCTAGGCACTCACTGATGTTTTTC |
| Human | Nlrp3 (d site)-F | CCATTATGCACTCCCAGCTTCAT |
| Human | Nlrp3 (d site)-R | CCAGGAGGTGAGTAAGTGTGTTG |
| Human | Nlrp3 (e site)-F | CCTCTTCCCTCACAAAAACAGAAGCA |
| Human | Nlrp3 (e site)-R | CGTTGATTACGGGGCTATGACATTG |
| Human | siRNA-Klf15-F | GACGTGAAGCTCAAGATCATT |
| Human | siRNA-Klf15-R | TGATCTTGAGCTTCACGTCTT |
| Human | siRNA-Fos-F | CCATCAGCCTGAGCCATTATT |
| Human | siRNA-Fos-R | TAATGGCTCAGGCTGATGGTT |
| Human | siRNA-*Ets1-*F | GGATCAAGCTCAAGAACAATT |
| Human | siRNA-*Ets1-*R | TTGTTCTTGAGCTTGATCCTT |
| Human | siRNA-*Egr1-*F | GGAAGTGTCTCAACGACATTT |
| Human | siRNA-*Egr1-R* | AATGTCGTTGAGACACTTCCT |
| Human | siRNA-Elf3-F | GGACTTCATCTACGAGAAATT |
| Human | siRNA-Elf3-R | TTTCTCGTAGATGAAGTCCTT |
| Human | KLF15-F | CGCGGATCCGCCACCATGGCGGCGGCGGCGGCG |
| Human | KLF15-R | CCGCTCGAGTCAGGCGCAGGTGGCGGCG |
| Human | Sirt6 (promoter)-F | CCGCTCGAGTGATGGTAAATAAGAAGGGC |
| Human | Sirt6 (promoter)-R | CCCAAGCTTTGAAAGTTTCCCTTGTTGAG |
| Human | Sirt6 (promoter) (4182863  ~4183063)-F | TGGGATGTTGAGCACCTGCCT |
| Human | Sirt6 (promoter) (4182863  ~4183063)-R | CAGGCGGCCGCCCCAGGG |
| Human | Sirt6 (promoter) (4184213  ~4184413)-F | CCTGGCTAGGACTCAGCACGG |
| Human | Sirt6 (promoter) (4184213  ~4184413)-R | GAGGCGCATGCGCCTTGCC |
| Human | Sirt6 (promoter) (4184363  ~4184563)-F | GGAAGACACCCACGATCTTCC |
| Human | Sirt6 (promoter) (4184363  ~4184563)-R | TGGTCACATGTTTGTGTCCAC |
| Human | Sirt6 (promoter-mutant)-F | TTAACCTAAGGGTTTCCCTTGTTGAG |
| Human | Sirt6 (promoter-mutant)-R | TTAAACCCTTAGTTGTGAGCCACTGCG |

**Tab. S4 Primary antibodies**

| Antibody | Manufacturer | Number | Applications |
| --- | --- | --- | --- |
| SIRT6 | abcam | ab289970 | IHC |
| SIRT6 | abcam | ab191385 | WB, IHC |
| NLRP3 | abcam | ab263899 | WB |
| NLRP3 | Proteintech | 68102-1-Ig | IHC |
| H3K9ac | Abclonal | A22565 | WB, IHC, CUT&Tag, CHIP |
| H3K56ac | Abclonal | A21107 | WB, IHC |
| KIM-1 | Invitrogen | MA5-28211 | WB, IF |
| CD3 | abcam | ab16669 | IHC |
| F4/80 | Proteintech | 29414-1-AP | IHC |
| α-SMA | abcam | ab5694 | WB, IHC |
| COL1A1 | CST | 72026 | WB, IHC |
| FN | abcam | ab2413 | WB, IHC |
| IL-1β | Abways | CY5087 | WB, IHC |
| Caspase-1 | abcam | ab179515 | WB |
| RNAP2 | Invitrogen | MA1-46093 | CHIP |
| IgG | Proteintech | 30000-0-AP | CHIP |
| Aquaporin 1 | abcam | ab168387 | IF |
| Nepherin | Servicebio | GB12343 | IF |

**Fig. S1 A** Correlation between proximal tubular *Sirt6* mRNA levels and blood urea nitrogen (BUN) in human cohorts (n = 36). **B** Negative correlation between proximal tubular *Sirt6* mRNA levels and HbA1c in human cohorts (n = 36). **C** Correlation between proximal tubular *Sirt6* mRNA levels and fasting blood glucose in human cohorts (n = 36). **D** Correlation between proximal tubular *Sirt6* mRNA levels and body mass index (BMI) in human cohorts (n = 36). **E** Urine albumin-to-creatinine ratio (UACR) in *db/m* versus *db/db* mice (n = 6/group). **F-G** Representative western blots and quantitative analysis of whole kidney showing SIRT6, H3K9ac, and H3K56ac protein levels (n = 6/group). **H** Relative mRNA expression of Sirtuin family members in renal TECs in two groups of mice (n = 6/group). **I-J** Immunofluorescence staining of kidney injury molecule-1 (KIM-1; red) with DAPI nuclear counterstain (blue) in renal sections (n = 6/group), scale bars, 50 µm. **K** UACR in mice fed a control diet (CD) or HFD/STZ treatment (n=6/group). **L-M** Representative western blots and quantitative analysis of whole kidney showing SIRT6, H3K9ac, and H3K56ac protein levels (n = 6/group). **N** Relative mRNA expression of Sirtuin family members in renal TECs in two groups of mice (n = 6/group). **O-P** Immunofluorescence staining of kidney injury molecule-1 (KIM-1; red) with DAPI nuclear counterstain (blue) in renal sections (n = 6/group), scale bars, 50 µm. **Q** Schematic diagram of experimental design. **R-S** SIRT6 protein levels in TECs across four experimental groups (n = 6/group). **T** Schematic diagram of experimental design. **U-V** SIRT6 protein levels in TECs across four experimental groups (n = 6/group). Data represent mean ± SEM. Two-way ANOVA with Tukey's multiple comparisons (**G, H, M, N**), Unpaired *t*-test (**E, J, K, P)**, One-way ANOVA with Tukey's post hoc analysis (**S, V**), Spearman's rank correlation (**A-D**). ^*^*p* < 0.05, ^**^*p* < 0.01, ^***^*p* < 0.001.

**Fig. S2 A** Generation strategy for TEC-specific *Sirt6* knockout mice. **B, C** Body weight and blood glucose levels in four groups (n = 6/group). **D, E** Western blot analysis of H3K9ac, H3K56ac in TECs from four experimental groups (n = 6/group). **F-G** IHC of H3K9ac (first row) and H3K56ac (second row) in renal tissues with quantitative analysis (n = 6/group), scale bars, 50 µm. All data represent mean ± SEM. Unpaired *t*-test (**B**); Two-way ANOVA with Tukey's multiple comparisons test (**E, G**). Significance levels: ^*^*p* < 0.05, ^**^*p* < 0.01, ^***^*p* < 0.001.

**Fig. S3 A-D** Fibrotic markers (α-SMA, FN, COL1A1) were quantified by Western blot (n = 6/group). **E** Gene Set Enrichment Analysis (GSEA) of top 10 pathways showing normalized enrichment scores (NES) and Z-scores. **F** Relative *Sirt6* mRNA levels in HK-2 cells under different treatment conditions (n = 6/group). **G, H** Western blot revealed significant alterations in SIRT6 expression and concomitant changes in H3K9ac levels under experimental conditions (n = 6/group). GAPDH served as loading control. Data represent mean ± SEM. Two-way ANOVA with Tukey's multiple comparisons test (**B, C, D, H**); One-way ANOVA with Tukey's post hoc analysis (**F**). Statistical significance: ^*^*p* < 0.05, ^**^*p* < 0.01, ^***^*p* < 0.001.

**Fig. S4 A** Quantitative comparison of differential H3K9ac peaks between genotypes. **B** Relative mRNA levels in TECs of four groups (n = 6/group). **C-D** IHC of NLRP3 in renal tissues with quantification (n = 6/group), scale bar, 50 µm. **E-I** ChIP-PCR analysis of H3K9ac binding across *Nlrp3* promoter regions (sites a-e). **J, K** Western blot analysis demonstrated that OSS_128167 treatment significantly altered H3K9ac levels in HK-2 cells (n = 6/group). **L-P** Pharmacological inhibition of NLRP3 by MCC950 markedly reduced expression of FN and NLRP3, cleaved Caspase-1, and mature IL-1β in HK-2 cells (n = 6/group). **Q** SIRT6 mRNA levels in HK-2 cells transfected with siRNA for 48 hours (normalized to controls). Data represent mean ± SEM. Unpaired *t*-test (**D, K**); Two-way ANOVA with Tukey's multiple comparisons test (**B, M, N, O, P, Q**). ^*^*p* < 0.05, ^**^*p* < 0.01, ^***^*p* < 0.001.

**Fig. S5** **A, B** IHC of SIRT6 and quantification (n = 6/group), scale bars, 50 µm. **C, D** Western blot quantification demonstrated altered H3K9ac levels in TECs (n = 6/group). **E, F** IHC of CD3 and quantification (n = 6/group), scale bars, 50 µm. **G, H** Fibrotic markers (α-SMA, COL1A1, FN) were quantified by Western blot (n = 6/group). **I-M** Composite analysis showing Masson's trichrome staining (first row), IHC of H3K9ac (second row), CD3 (third row), and immunofluorescence of KIM-1(fourth row) with quantification (n = 6/group), scale bars, 50 µm. Data represent mean ± SEM. Unpaired *t*-test (**B, D**); Two-way ANOVA with Tukey's multiple comparisons (**F, H, K, L, M**), Kruskal-Wallis test with Dunn's correction (**J**). Significance levels: ^*^*p* < 0.05, ^**^*p* < 0.01, ^***^*p* < 0.001.

**Fig. S6- S10 Western blots**
