# Supplementary material for: Mitigation of renal tubular injury by SIRT6 may improve individual outcomes in diabetic kidney disease-potential mechanisms involving epigenetic repression of inflammatory responses
Source: J Adv Res. 2025 Oct 10;85:281–94. doi: 10.1016/j.jare.2025.10.008 (PMC13316547; doi:10.1016/j.jare.2025.10.008)

Fig. S1

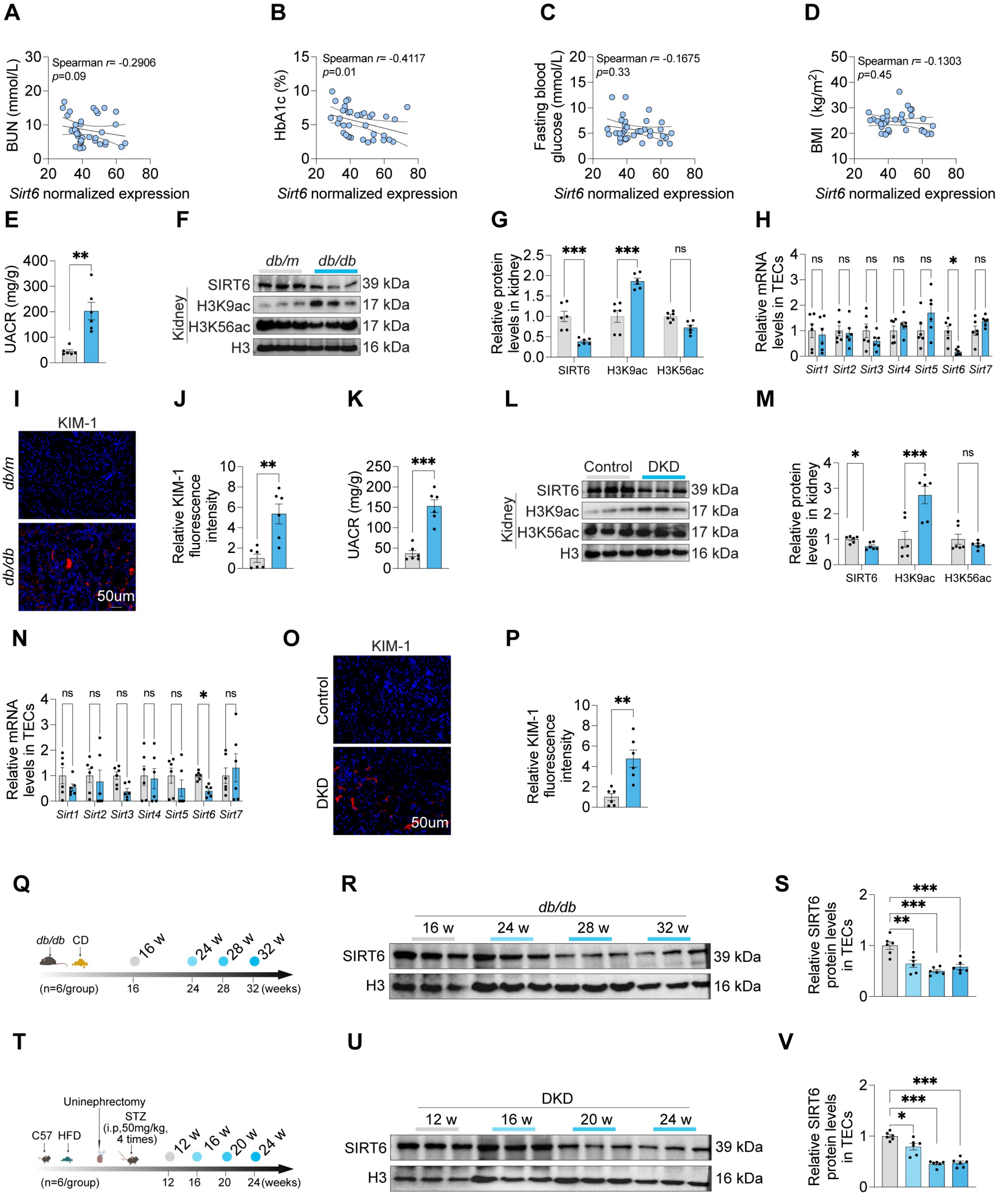
A
Spearman r= -0.2906
p=0.09
BUN (mmol/L)
Sirt6 normalized expression
B
Spearman r= -0.4117
p=0.01
HbA1c (%)
Sirt6 normalized expression
C
Spearman r= -0.1675
p=0.33
Fasting blood glucose (mmol/L)
Sirt6 normalized expression
D
Spearman r= -0.1303
p=0.45
BMI (kg/m2)
Sirt6 normalized expression
E
UACR (mg/g)
**
F
db/m
db/db
Kidney
SIRT6
H3K9ac
H3K56ac
H3
39 kDa
17 kDa
17 kDa
16 kDa
G
Relative protein levels in kidney
***
***
ns
SIRT6
H3K9ac
H3K56ac
H
Relative mRNA levels in TECs
ns
ns
ns
ns
ns
*
ns
Sirt1
Sirt2
Sirt3
Sirt4
Sirt5
Sirt6
Sirt7
I
KIM-1
db/m
db/db
50um
J
Relative KIM-1 fluorescence intensity
**
K
UACR (mg/g)
***
L
Control
DKD
Kidney
SIRT6
H3K9ac
H3K56ac
H3
39 kDa
17 kDa
17 kDa
16 kDa
M
Relative protein levels in kidney
*
***
ns
SIRT6
H3K9ac
H3K56ac
N
Relative mRNA levels in TECs
ns
ns
ns
ns
ns
*
ns
Sirt1
Sirt2
Sirt3
Sirt4
Sirt5
Sirt6
Sirt7
O
KIM-1
Control
DKD
50um
P
Relative KIM-1 fluorescence intensity
**
Q
db/db
CD
16 w
24 w
28 w
32 w
(n=6/group)
16
24
28
32 (weeks)
R
db/db
16 w
24 w
28 w
32 w
SIRT6
H3
39 kDa
16 kDa
S
Relative SIRT6 protein levels in TECs
**
***
***
T
Uninephrectomy
C57
HFD
STZ (i.p,50mg/kg, 4 times)
12 w
16 w
20 w
24 w
(n=6/group)
12
16
20
24 (weeks)
U
DKD
12 w
16 w
20 w
24 w
SIRT6
H3
39 kDa
16 kDa
V
Relative SIRT6 protein levels in TECs
*
***
***

Fig. S1 **A** Correlation between proximal tubular *Sirt6* mRNA levels and blood urea nitrogen (BUN) in human cohorts (n = 36). **B** Negative correlation between proximal tubular *Sirt6* mRNA levels and HbA1c in human cohorts (n = 36). **C** Correlation between proximal tubular *Sirt6* mRNA levels and fasting blood glucose in human cohorts (n = 36). **D** Correlation between proximal tubular *Sirt6* mRNA levels and body mass index (BMI) in human cohorts (n = 36). **E** Urine albumin-to-creatinine ratio (UACR) in *db/m* versus *db/db* mice (n = 6/group). **F-G** Representative western blots and quantitative analysis of whole kidney showing SIRT6, H3K9ac, and H3K56ac protein levels (n = 6/group). **H** Relative mRNA expression of Sirtuin family members in renal TECs in two groups of mice (n = 6/group). **I-J** Immunofluorescence staining of kidney injury molecule-1 (KIM-1; red) with DAPI nuclear counterstain (blue) in renal sections (n = 6/group), scale bars, 50 µm. **K** UACR in mice fed a control diet (CD) or HFD/STZ treatment (n=6/group). **L-M** Representative western blots and quantitative analysis of whole kidney showing SIRT6, H3K9ac, and H3K56ac protein levels (n = 6/group). **N** Relative mRNA expression of Sirtuin family members in renal TECs in two groups of mice (n = 6/group). **O-P** Immunofluorescence staining of kidney injury molecule-1 (KIM-1; red) with DAPI nuclear counterstain (blue) in renal sections (n = 6/group), scale bars, 50 µm. **Q** Schematic diagram of experimental design. **R-S** SIRT6 protein levels in TECs across four experimental groups (n = 6/group). **T** Schematic diagram of experimental design. **U-V** SIRT6 protein levels in TECs across four experimental groups (n = 6/group). Data represent mean ± SEM. Two-way ANOVA with Tukey's multiple comparisons (**G, H, M, N**), Unpaired *t*-test (**E, J, K, P)**, One-way ANOVA with Tukey's post hoc analysis (**S, V**), Spearman's rank correlation (**A-D**). $^{*}p < 0.05$, $^{**}p < 0.01$, $^{***}p < 0.001$.

Fig. S2

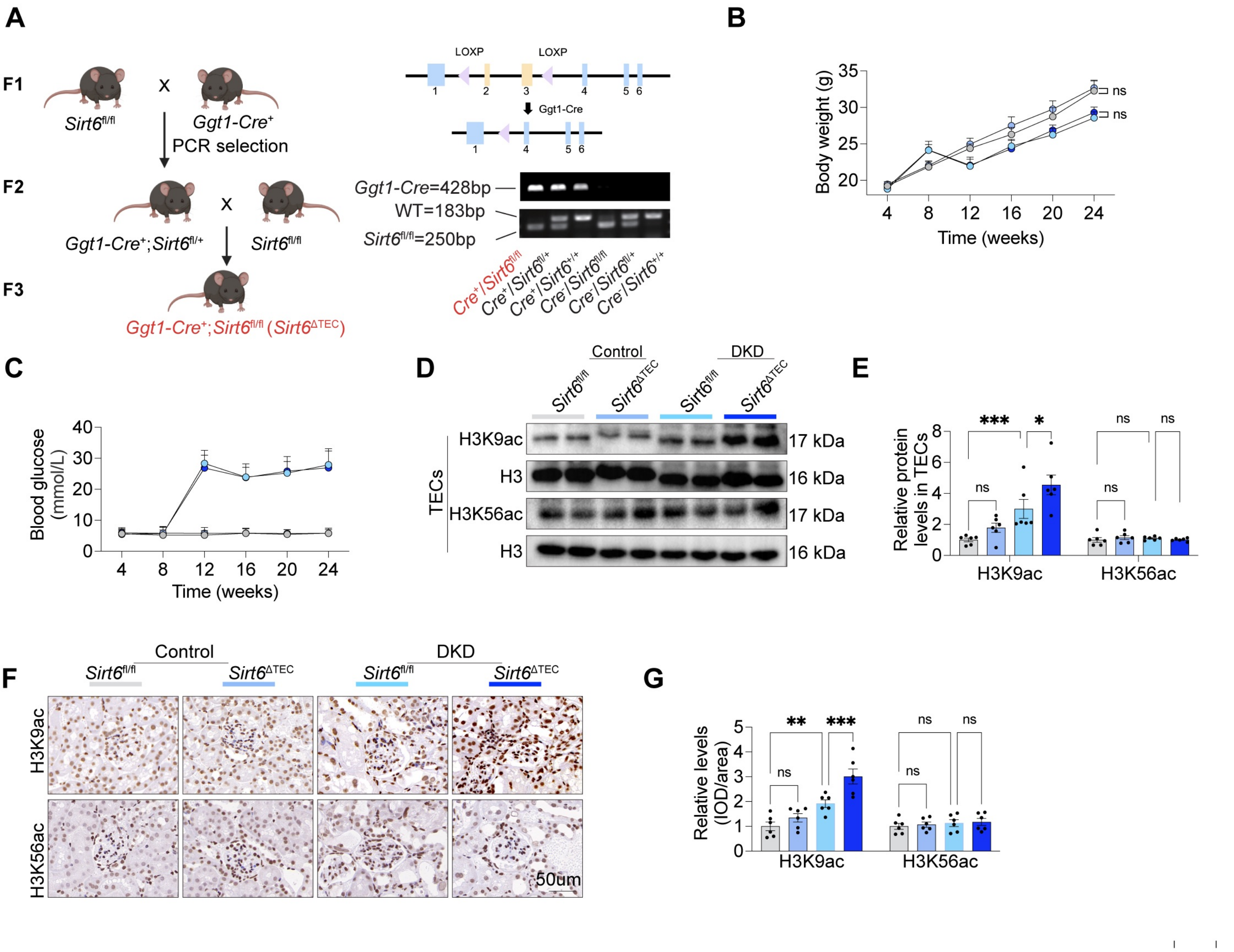

A
F1
F2
F3
Sirt6fl/fl
Ggt1-Cre+
PCR selection
Ggt1-Cre+;Sirt6fl/+
Sirt6fl/fl
Ggt1-Cre+;Sirt6fl/fl (Sirt6ΔTEC)
LOXP
Ggt1-Cre
Ggt1-Cre=428bp
WT=183bp
Sirt6fl/fl=250bp
Cre+/Sirt6fl/fl
Cre+/Sirt6fl/+
Cre+/Sirt6+/+
Cre-/Sirt6fl/fl
Cre-/Sirt6fl/+
Cre-/Sirt6+/+
B
Body weight (g)
Time (weeks)
ns
C
Blood glucose (mmol/L)
Time (weeks)
D
Control
DKD
Sirt6fl/fl
Sirt6ΔTEC
TECs
H3K9ac
H3
H3K56ac
17 kDa
16 kDa
E
Relative protein levels in TECs
H3K9ac
H3K56ac
F
H3K9ac
H3K56ac
50um
G
Relative levels (IOD/area)

Fig. S2 A Generation strategy for TEC-specific *Sirt6* knockout mice. **B, C** Body weight and blood glucose levels in four groups (n = 6/group). **D, E** Western blot analysis of H3K9ac, H3K56ac in TECs from four experimental groups (n = 6/group). **F-G** IHC of H3K9ac (first row) and H3K56ac (second row) in renal tissues with quantitative analysis (n = 6/group), scale bars, 50 µm. All data represent mean ± SEM. Unpaired *t*-test (**B**); Two-way ANOVA with Tukey's multiple comparisons test (**E, G**). Significance levels: $^{*}p < 0.05$, $^{**}p < 0.01$, $^{***}p < 0.001$.

Fig. S3

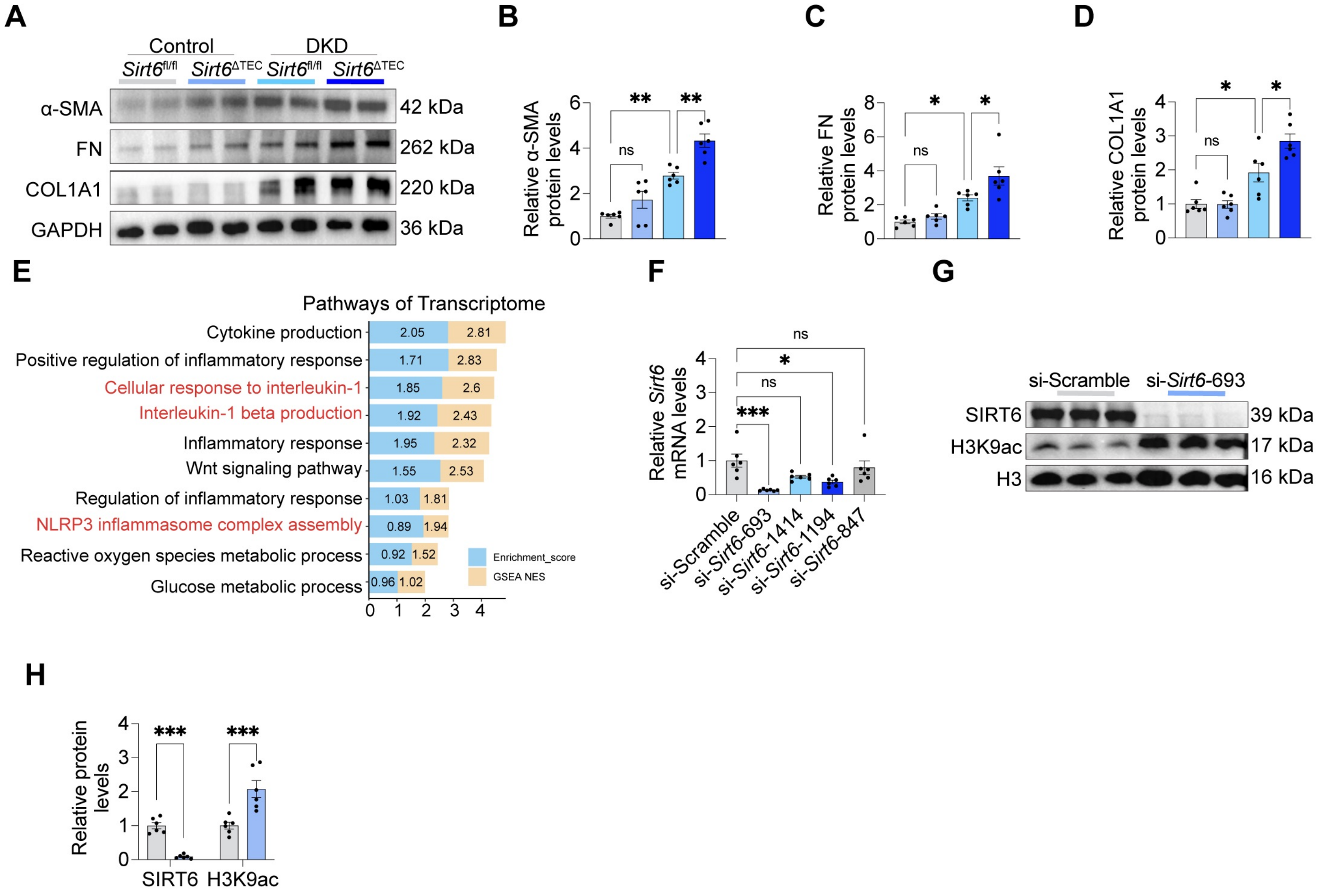
A
Control
DKD
Sirt6fl/fl
Sirt6ΔTEC
α-SMA
FN
COL1A1
GAPDH
42 kDa
262 kDa
220 kDa
36 kDa
B
Relative α-SMA protein levels
C
Relative FN protein levels
D
Relative COL1A1 protein levels
E
Pathways of Transcriptome
Cytokine production
Positive regulation of inflammatory response
Cellular response to interleukin-1
Interleukin-1 beta production
Inflammatory response
Wnt signaling pathway
Regulation of inflammatory response
NLRP3 inflammasome complex assembly
Reactive oxygen species metabolic process
Glucose metabolic process
Enrichment_score
GSEA NES
F
Relative Sirt6 mRNA levels
si-Scramble
si-Sirt6-693
si-Sirt6-1414
si-Sirt6-1194
si-Sirt6-847
G
si-Scramble
si-Sirt6-693
SIRT6
H3K9ac
H3
39 kDa
17 kDa
16 kDa
H
Relative protein levels
SIRT6
H3K9ac

Fig. S3 A-D Fibrotic markers (α-SMA, FN, COL1A1) were quantified by Western blot (n = 6/group). **E** Gene Set Enrichment Analysis (GSEA) of top 10 pathways showing normalized enrichment scores (NES) and Z-scores. **F** Relative *Sirt6* mRNA levels in HK-2 cells under different treatment conditions (n = 6/group). **G, H** Western blot revealed significant alterations in SIRT6 expression and concomitant changes in H3K9ac levels under experimental conditions (n = 6/group). GAPDH served as loading control. Data represent mean ± SEM. Two-way ANOVA with Tukey's multiple comparisons test (**B, C, D, H**); One-way ANOVA with Tukey's post hoc analysis (**F**). Statistical significance: $^{*}p < 0.05$, $^{**}p < 0.01$, $^{***}p < 0.001$.

Fig. S4

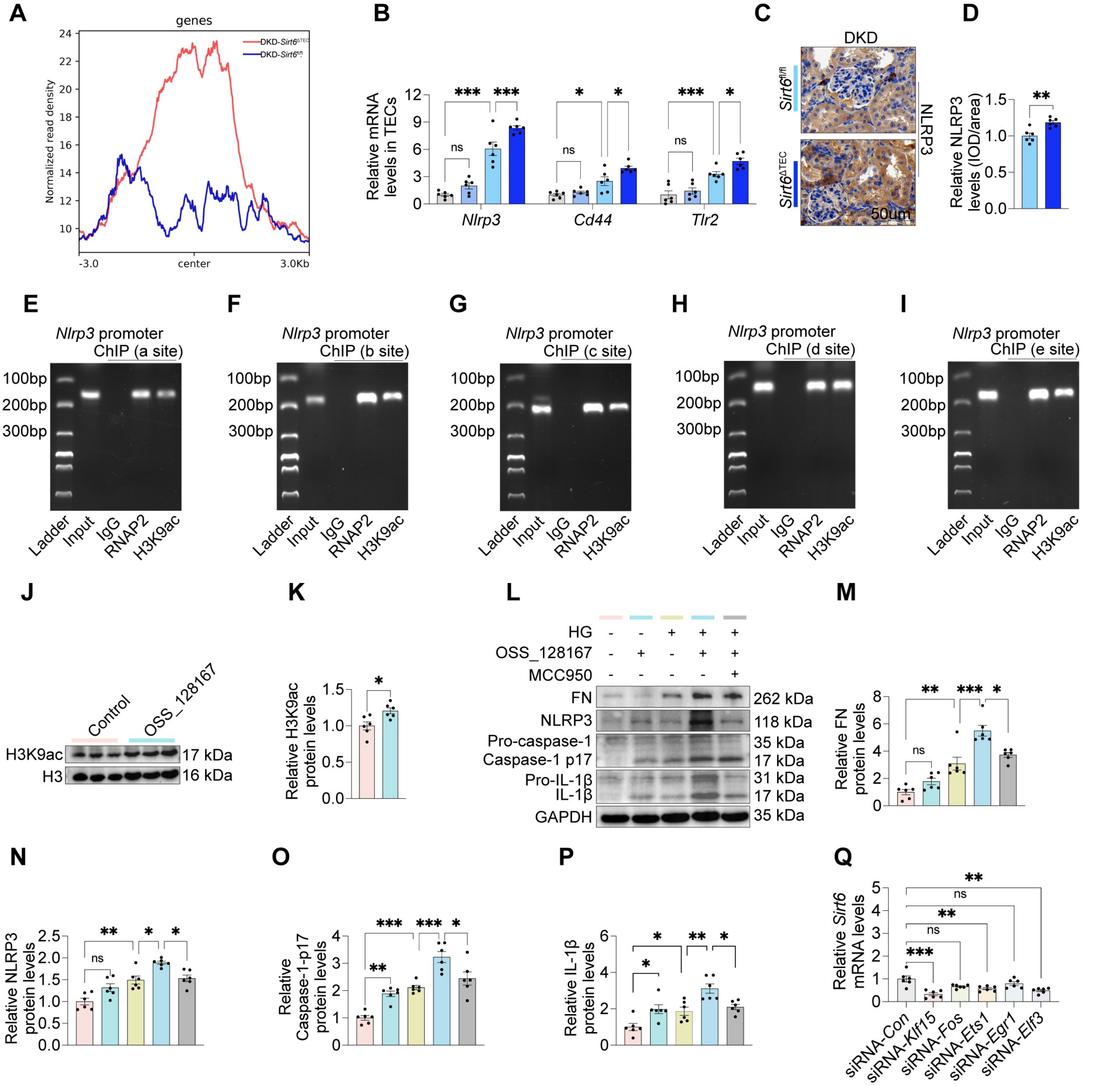
A
genes
DKD-Sirt6ΔTEC
DKD-Sirt6fl/fl
Normalized read density
-3.0
center
3.0Kb
B
Relative mRNA levels in TECs
Nlrp3
Cd44
Tlr2
ns
C
DKD
Sirt6fl/fl
Sirt6ΔTEC
NLRP3
50um
D
Relative NLRP3 levels (IOD/area)
E
Nlrp3 promoter ChIP (a site)
F
Nlrp3 promoter ChIP (b site)
G
Nlrp3 promoter ChIP (c site)
H
Nlrp3 promoter ChIP (d site)
I
Nlrp3 promoter ChIP (e site)
100bp
200bp
300bp
Ladder
Input
IgG
RNAP2
H3K9ac
J
Control
OSS_128167
H3K9ac
H3
17 kDa
16 kDa
K
Relative H3K9ac protein levels
L
HG
OSS_128167
MCC950
FN
NLRP3
Pro-caspase-1
Caspase-1 p17
Pro-IL-1β
IL-1β
GAPDH
262 kDa
118 kDa
35 kDa
17 kDa
31 kDa
17 kDa
35 kDa
M
Relative FN protein levels
N
Relative NLRP3 protein levels
O
Relative Caspase-1-p17 protein levels
P
Relative IL-1β protein levels
Q
Relative Sirt6 mRNA levels
siRNA-Con
siRNA-Klf15
siRNA-Fos
siRNA-Ets1
siRNA-Egr1
siRNA-Elf3

Fig. S4 A Quantitative comparison of differential H3K9ac peaks between genotypes. **B** Relative mRNA levels in TECs of four groups (n = 6/group). **C-D** IHC of NLRP3 in renal tissues with quantification (n = 6/group), scale bar, 50 μm. **E-I** ChIP-PCR analysis of H3K9ac binding across *Nlrp3* promoter regions (sites a-e). **J, K** Western blot analysis demonstrated that OSS_128167 treatment significantly altered H3K9ac levels in HK-2 cells (n = 6/group). **L-P** Pharmacological inhibition of NLRP3 by MCC950 markedly reduced expression of FN and NLRP3, cleaved Caspase-1, and mature IL-1β in HK-2 cells (n = 6/group). **Q** SIRT6 mRNA levels in HK-2 cells transfected with siRNA for 48 hours (normalized to controls). Data represent mean ± SEM. Unpaired *t*-test (**D, K**); Two-way ANOVA with Tukey's multiple comparisons test (**B, M, N, O, P, Q**). $^{*}p < 0.05$, $^{**}p < 0.01$, $^{***}p < 0.001$.

Fig. S5

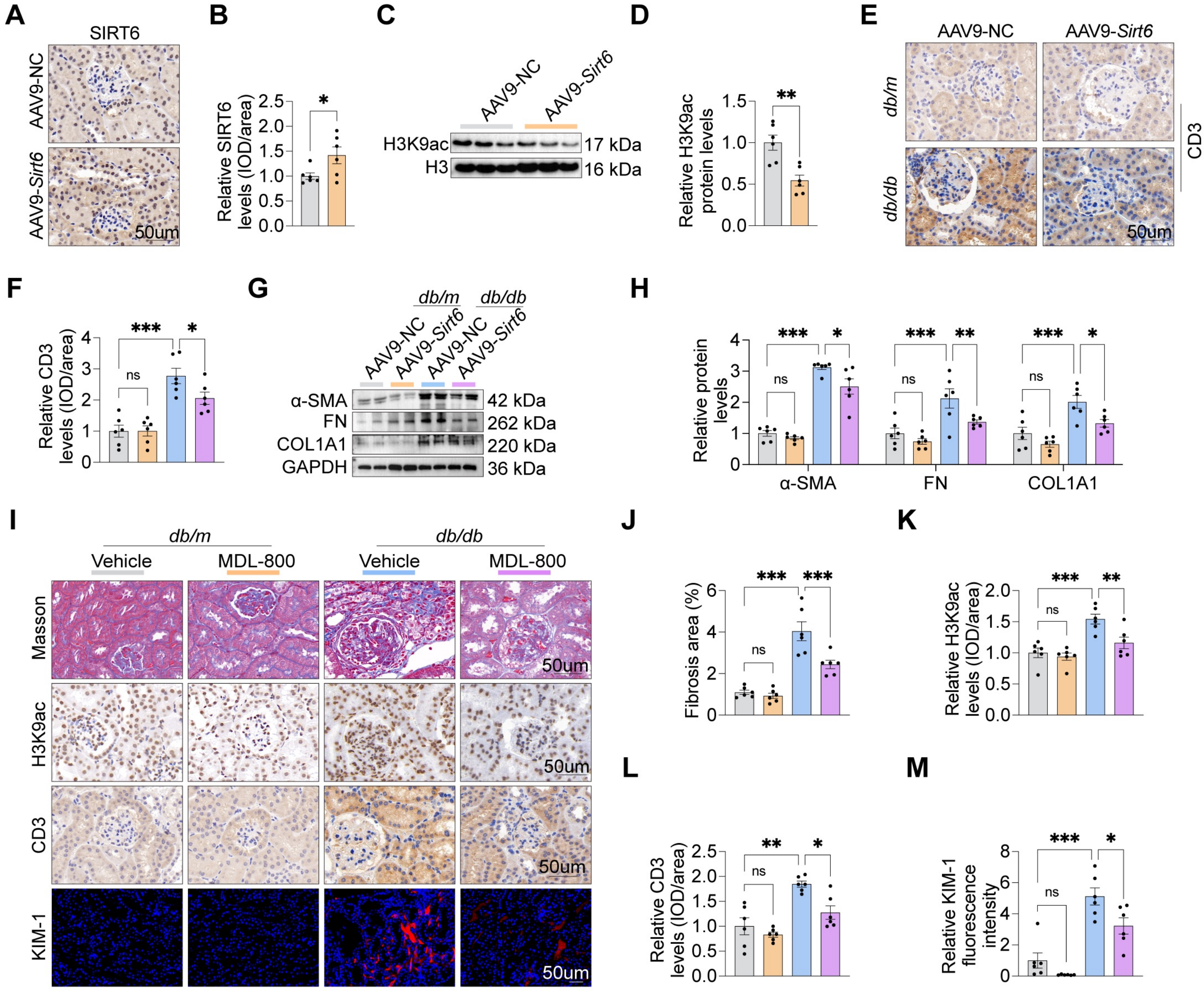

A
SIRT6
AAV9-NC
AAV9-Sirt6
50um
B
Relative SIRT6 levels (IOD/area)
*
C
AAV9-NC
AAV9-Sirt6
H3K9ac
H3
17 kDa
16 kDa
D
Relative H3K9ac protein levels
**
E
AAV9-NC
AAV9-Sirt6
db/m
db/db
CD3
50um
F
Relative CD3 levels (IOD/area)
G
db/m
db/db
AAV9-NC
AAV9-Sirt6
α-SMA
FN
COL1A1
GAPDH
42 kDa
262 kDa
220 kDa
36 kDa
H
Relative protein levels
α-SMA
FN
COL1A1
I
db/m
db/db
Vehicle
MDL-800
Masson
H3K9ac
CD3
KIM-1
50um
J
Fibrosis area (%)
K
Relative H3K9ac levels (IOD/area)
L
Relative CD3 levels (IOD/area)
M
Relative KIM-1 fluorescence intensity

Fig. S5 A, B IHC of SIRT6 and quantification (n = 6/group), scale bars, 50 μm. **C, D** Western blot quantification demonstrated altered H3K9ac levels in TECs (n = 6/group). **E, F** IHC of CD3 and quantification (n = 6/group), scale bars, 50 μm. **G, H** Fibrotic markers (α-SMA, COL1A1, FN) were quantified by Western blot (n = 6/group). **I-M** Composite analysis showing Masson's trichrome staining (first row), IHC of H3K9ac (second row), CD3 (third row), and immunofluorescence of KIM-1(fourth row) with quantification (n = 6/group), scale bars, 50 μm. Data represent mean ± SEM. Unpaired *t*-test (**B, D**); Two-way ANOVA with Tukey's multiple comparisons (**F, H, K, L, M**), Kruskal-Wallis test with Dunn's correction (**J**). Significance levels: $^{*}p < 0.05$, $^{**}p < 0.01$, $^{***}p < 0.001$.

Fig. S6

Fig. 1(N)

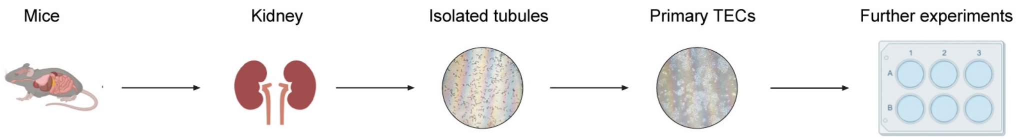

Fig. S1(D)

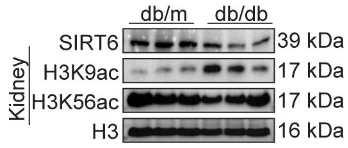

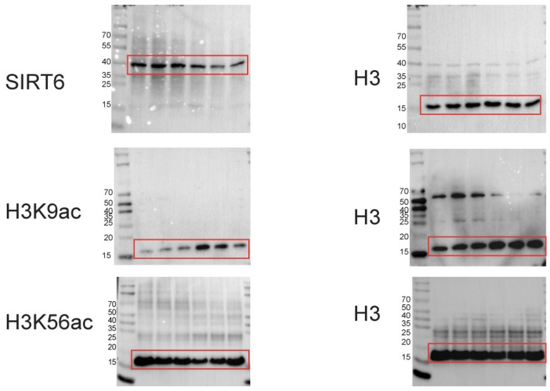

Fig. 1(O)

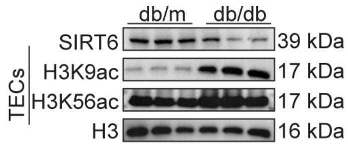

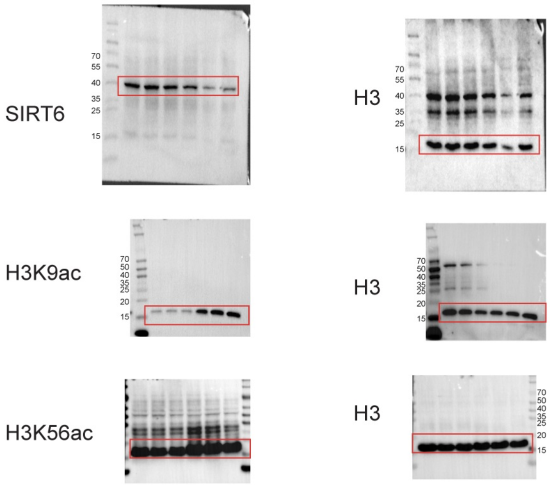

Fig. S1(J)

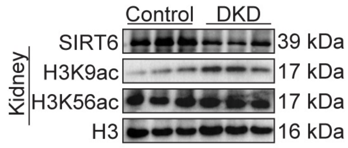

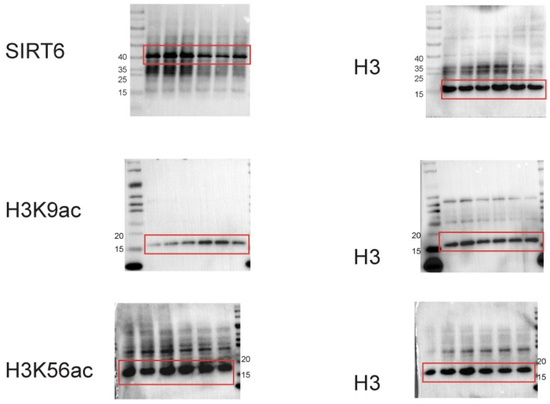

Fig. S1(T)

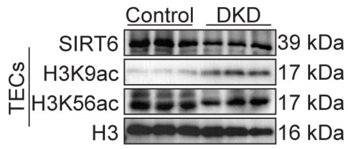

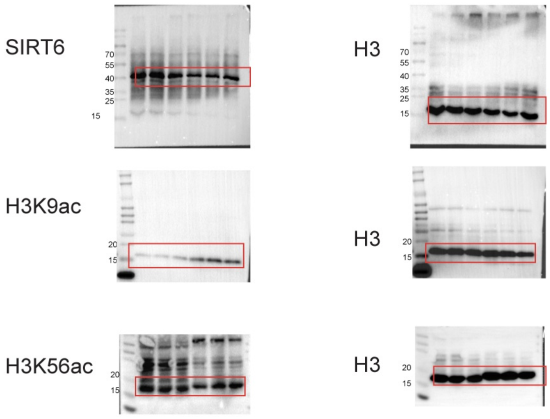

Fig. S7

Fig. S1(O)

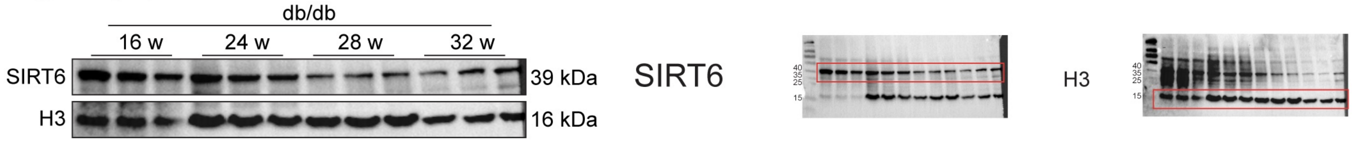

Fig. S1(R)

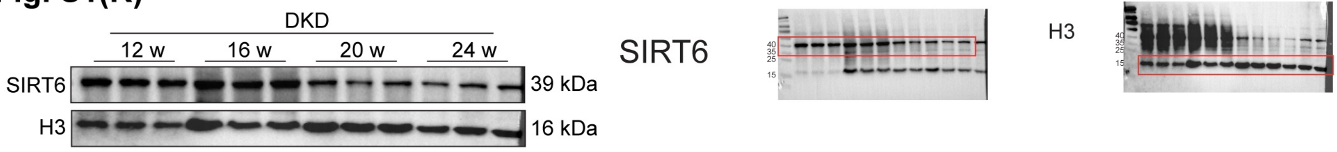

Fig. 2(C)

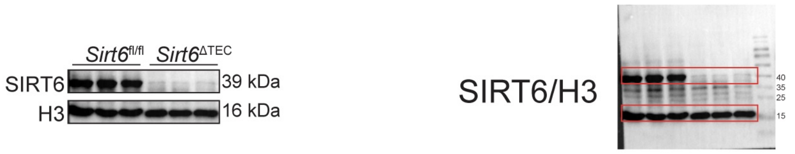

Fig. 2(Q)

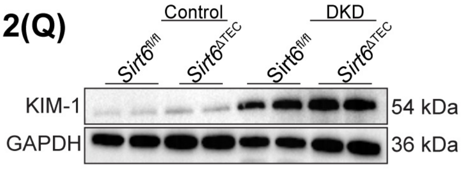

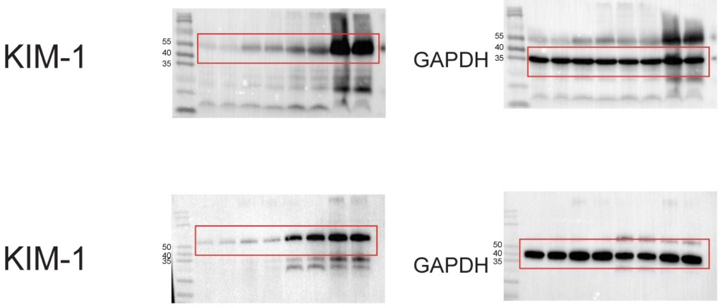

Fig. S2(D)

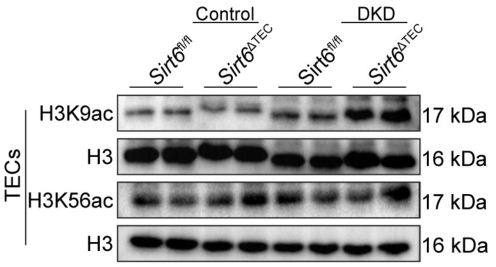

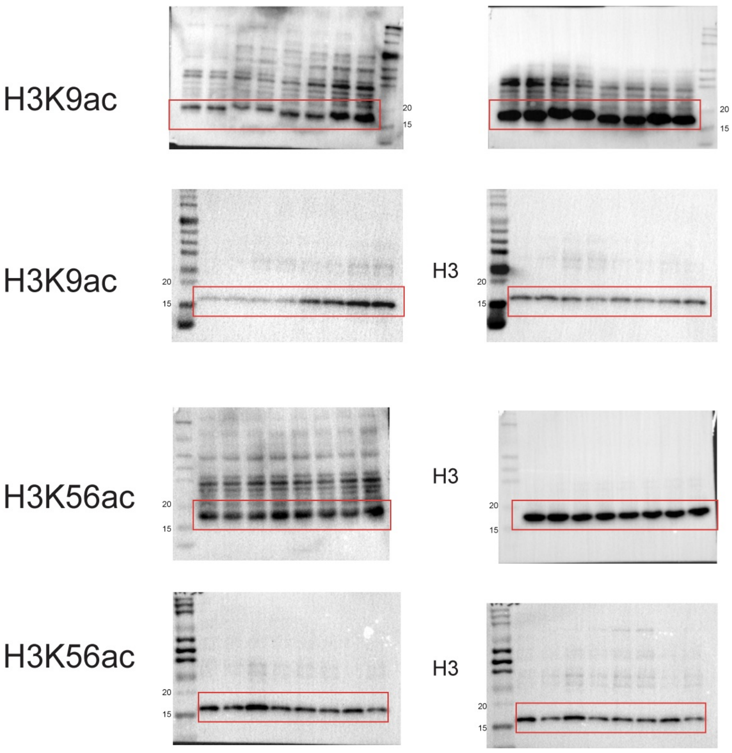

Fig. S8

Fig. 3(L)

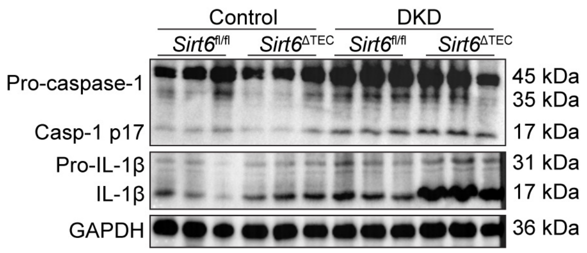

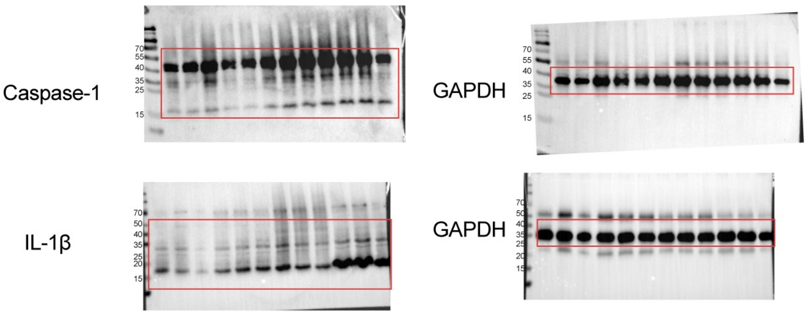

Fig. S3(G)

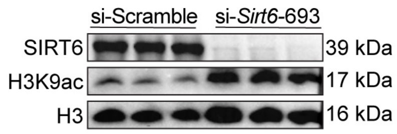

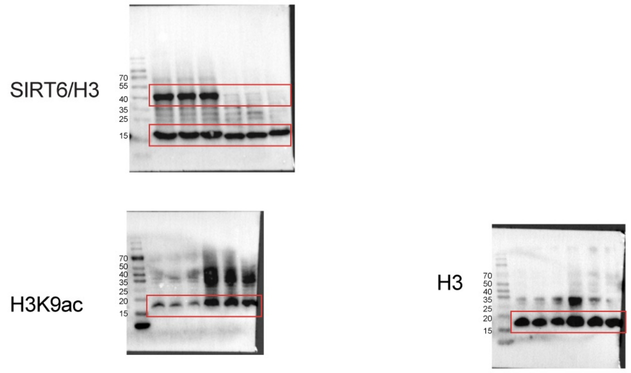

Fig. 3(N)

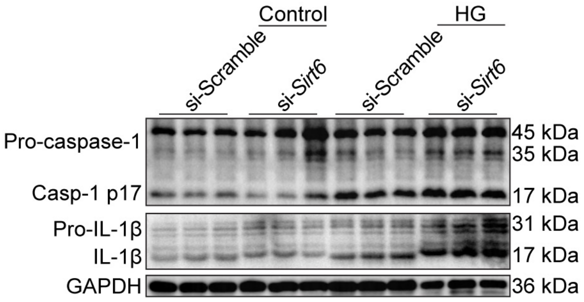

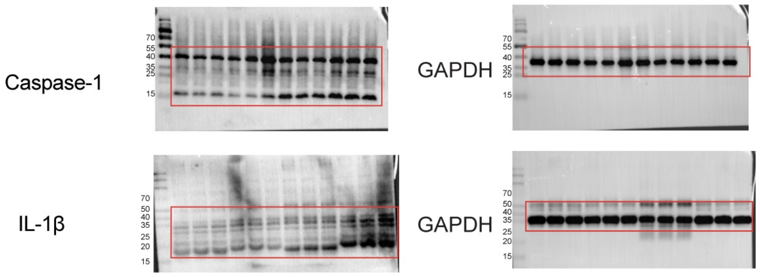

Fig. S3(A)

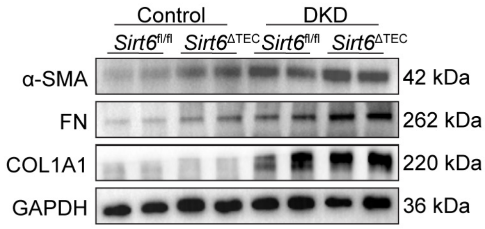

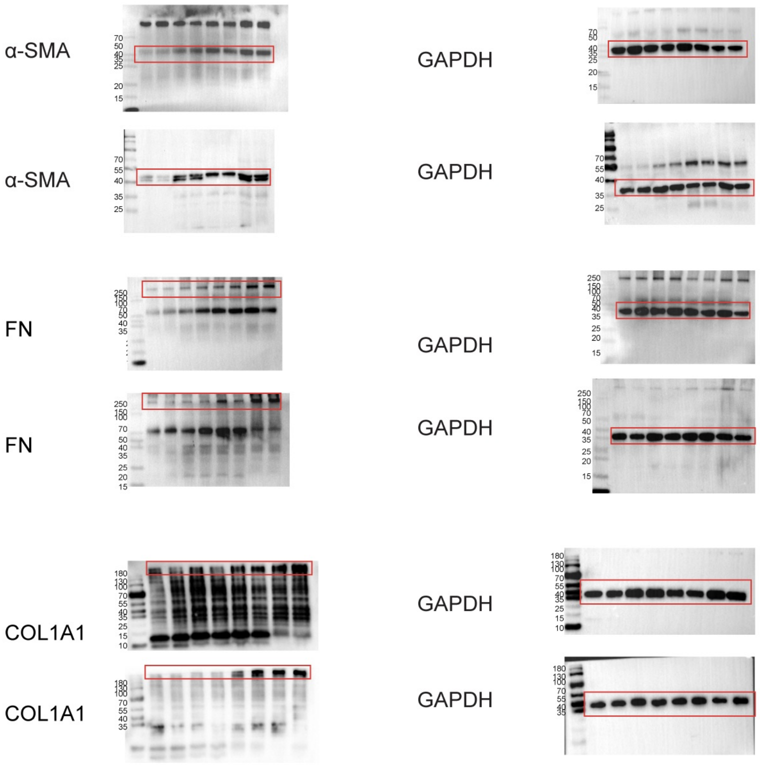

Fig. S9

Fig. S4(H)

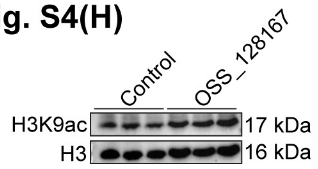

Fig. S4(J)

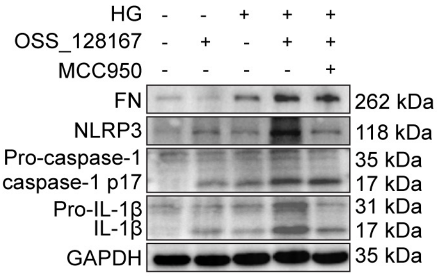

Fig. 4 (O)

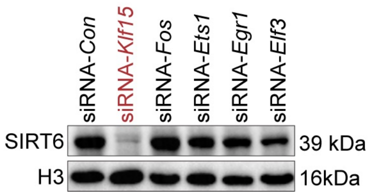

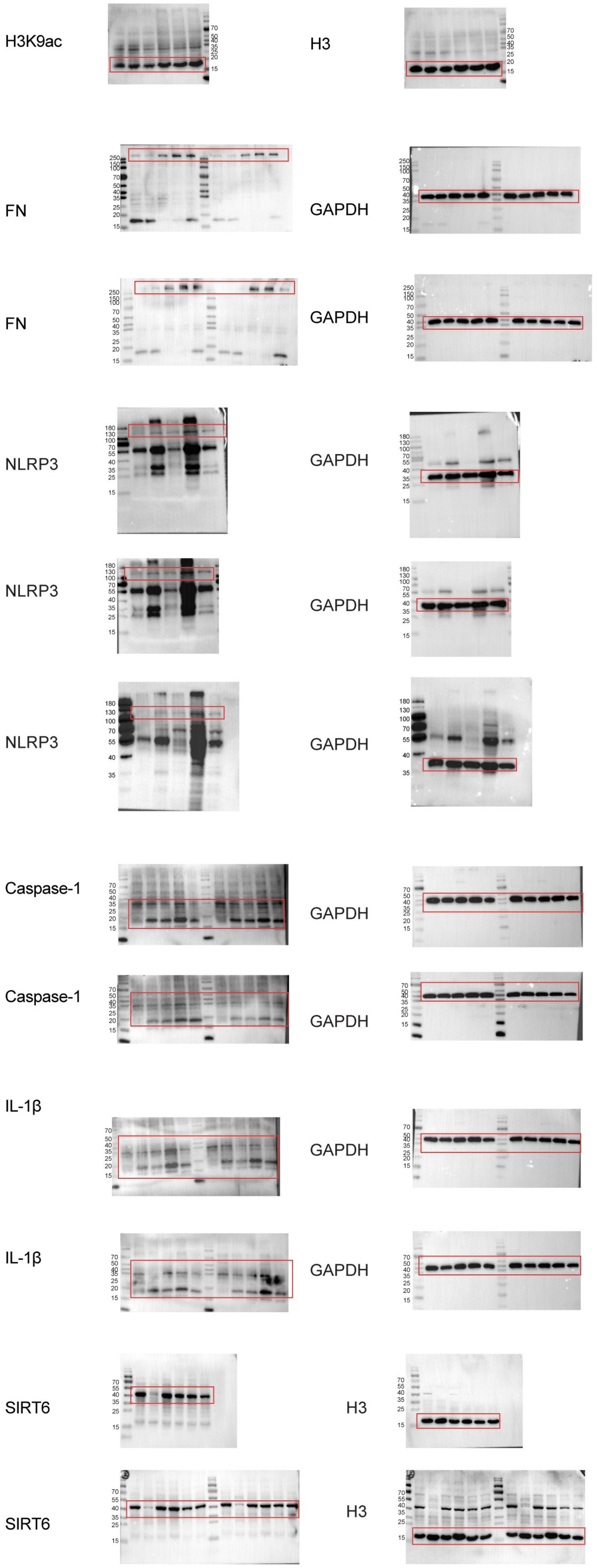

Fig. S10

Fig. 5(M)

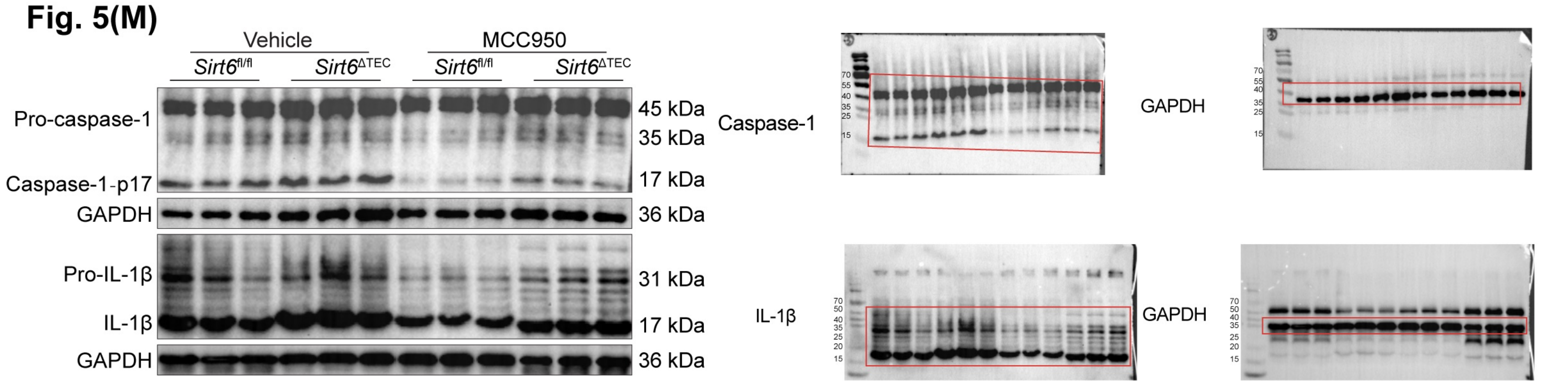

Fig. 6(B)

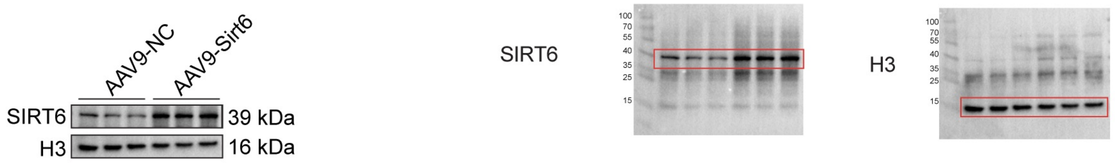

Fig. S6(C)

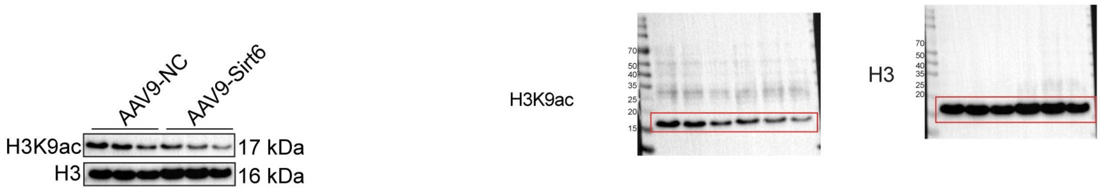

Fig. S6(G)

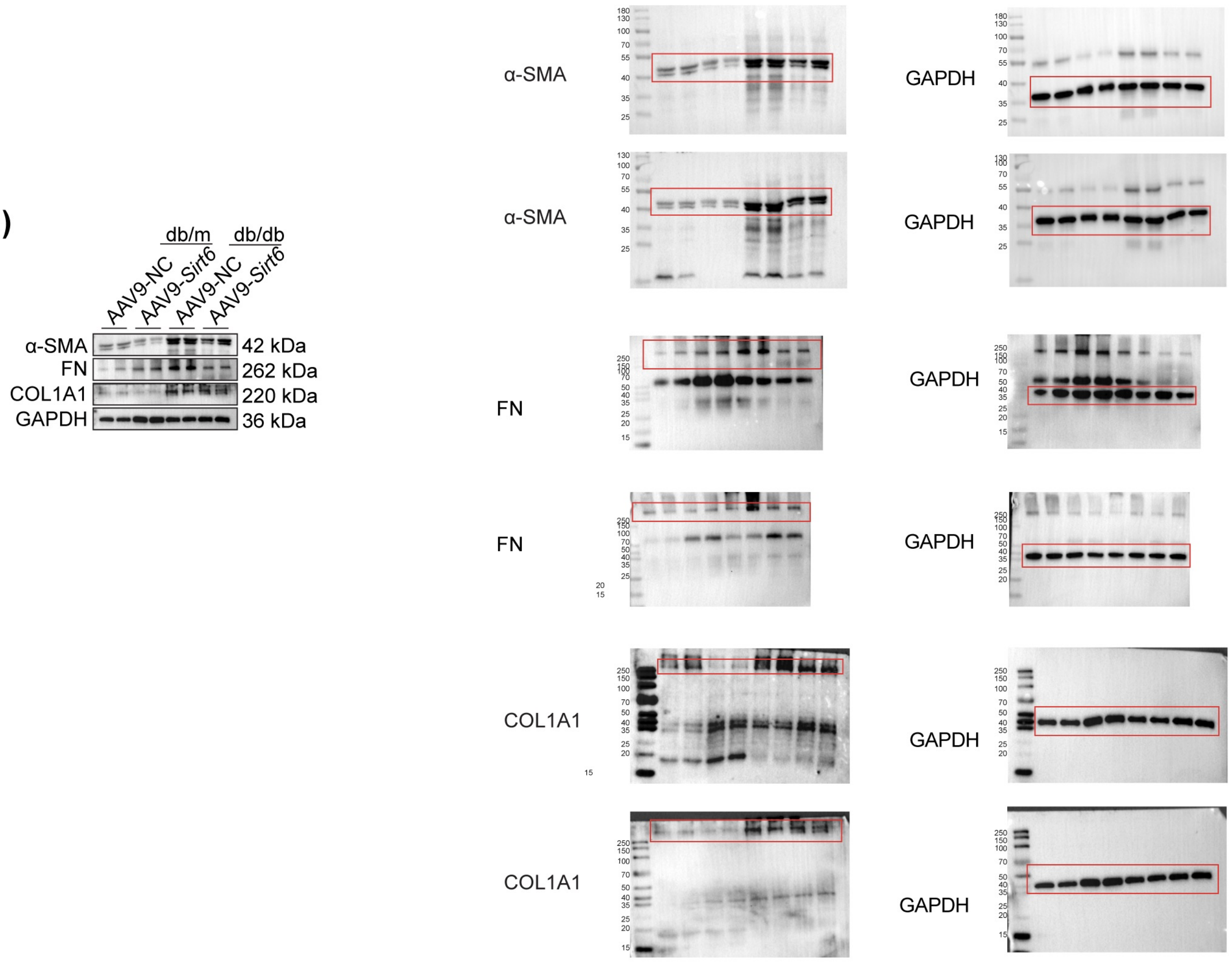

Supplement: Supplementary Data 1 [file mmc2.pdf]
